# Odontocete spatial patterns and temporal drivers of detection at sites in the Hawaiian islands

**DOI:** 10.1002/ece3.9688

**Published:** 2023-01-06

**Authors:** Morgan A. Ziegenhorn, John A. Hildebrand, Erin M. Oleson, Robin W. Baird, Sean M. Wiggins, Simone Baumann‐Pickering

**Affiliations:** ^1^ Scripps Institution of Oceanography University of California San Diego La Jolla California USA; ^2^ NOAA Fisheries Pacific Islands Fisheries Science Center Honolulu Hawaii USA; ^3^ Cascadia Research Collective Olympia Washington USA

**Keywords:** ecology, Hawaiian islands, marine mammal, odontocetes, species composition, temporal patterns

## Abstract

Successful conservation and management of marine top predators rely on detailed documentation of spatiotemporal behavior. For cetacean species, this information is key to defining stocks, habitat use, and mitigating harmful interactions. Research focused on this goal is employing methodologies such as visual observations, tag data, and passive acoustic monitoring (PAM) data. However, many studies are temporally limited or focus on only one or few species. In this study, we make use of an existing long‐term (2009–2019), labeled PAM data set to examine spatiotemporal patterning of at least 10 odontocete (toothed whale) species in the Hawaiian Islands using compositional analyses and modeling techniques. Species composition differs among considered sites, and this difference is robust to seasonal movement patterns. Temporally, hour of day was the most significant predictor of detection across species and sites, followed by season, though patterns differed among species. We describe long‐term trends in species detection at one site and note that they are markedly similar for many species. These trends may be related to long‐term, underlying oceanographic cycles that will be the focus of future study. We demonstrate the variability of temporal patterns even at relatively close sites, which may imply that wide‐ranging models of species presence are missing key fine‐scale movement patterns. Documented seasonal differences in detection also highlights the importance of considering season in survey design both regionally and elsewhere. We emphasize the utility of long‐term, continuous monitoring in highlighting temporal patterns that may relate to underlying climatic states and help us predict responses to climate change. We conclude that long‐term PAM records are a valuable resource for documenting spatiotemporal patterns and can contribute many insights into the lives of top predators, even in highly studied regions such as the Hawaiian Islands.

## Graphical abstract

In this study, we make use of an existing long‐term, labeled PAM data set to examine spatiotemporal patterning of at least 10 odontocete (toothed whale) species in the Hawaiian Islands using compositional analyses and modeling techniques. We demonstrate the variability of temporal patterns even at relatively close sites, which may imply that wide‐ranging models of species presence are missing key fine‐scale movement patterns.

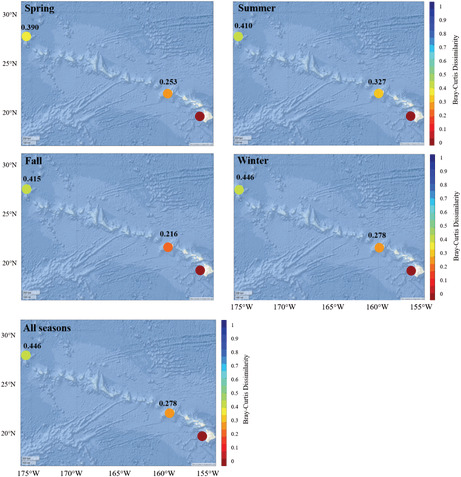

## 1 INTRODUCTION

Documenting spatiotemporal patterns of species presence is a crucial part of the conservation and management of marine top predators. Detailed spatial information about species presence often aids in the definition of new or distinct populations as well as the understanding of habitat use and movement patterns (e.g., Baird et al., 2010; Scofield et al., 2011; Sequeira et al., 2018). Continuous temporal data facilitate description of key patterns in animal activity, which is crucial for understanding foraging strategies and mitigating harmful anthropogenic interactions (Forney et al., 2011; Jones et al., 2019; Soldevilla et al., 2011). Characterization of spatiotemporal patterns in odontocete (toothed whale) species presence may also facilitate the creation and comparison of habitat models (e.g., Kanaji et al., 2017; Seger & Miksis‐Olds, 2020). Studies focused on these goals provide valuable baselines to which additional work can be compared. This allows scientists to monitor populations and detect changes over time that may be related to underlying changes in oceanographic and climate patterns or anthropogenic activity.

In the Hawaiian Islands, odontocetes are one of the most speciose groups of marine top predators, with at least 18 species frequenting or residing in this region (Baird, Webster, et al., 2013). Factors such as quality of environment (Schmelzer, 2000), foraging opportunities related to island‐associated prey (Abecassis et al., 2015), and movement to or from important geographical features (Thorne et al., 2012) may influence fine‐scale movements of these animals. Much of what is known about these species' general distribution has been documented in National Oceanic and Atmospheric Administration (NOAA) stock assessments. These assessments are largely derived from multi‐month line‐transect visual surveys (e.g., Bradford et al., 2021), tag data (e.g., Baird et al., 2010) and regional studies involving small boat surveys (e.g., Baird, Webster, et al., 2013). Passive acoustic monitoring (PAM) has also been used to study these animals but has often been limited by the difficulty of classifying multiple species using the detected sounds, focusing instead on only one or a few species of odontocetes whose calls are well described (Soldevilla et al., 2011; Squires et al., 2014; Wang et al., 2015; Wirth & Warren, 2017). These methods of study have resulted in an overall base of knowledge that contains extensive information for some species but is compiled from disparate records, often with limited spatial or temporal coverage.

A PAM data set from the Hawaiian archipelago presents a unique opportunity to examine odontocete fine‐scale temporal patterns at several sites over a longer time frame (2009–2019) than most other studies. These recordings include data from two subsites at a remote island in the Northwestern Hawaiian Islands (Manawai, otherwise known as Pearl and Hermes Reef), where records of odontocete spatiotemporal trends are limited compared to the Main Hawaiian Islands. A recent study that applied a machine learning toolkit to this data set resulted in labeled data for eight groupings of odontocetes (Ziegenhorn et al., 2022). These groupings included five species‐specific labels: false killer whale (*Pseudorca crassidens*), rough‐toothed dolphin (*Steno bredanensis*), short‐finned pilot whale (*Globicephala macrorhyncus*), Blainville's beaked whale (*Mesoplodon densirostris*), and Cuvier's beaked whale (*Ziphius cavirostris*). Additional genus or group‐level labels were stenellid dolphins (including some mixture of pantropical spotted dolphin (*Stenella attenuata*), striped dolphin (*S. coeruleoalba*), and spinner dolphin (*S. longirostris*)), *Kogia* spp. (primarily dwarf sperm whale (*K. sima*)), but potentially containing detections of pygmy sperm whale (*K. breviceps*) based on sighting records (Baird et al., 2021), and a type representing an unknown mixture of common bottlenose dolphin (*Tursiops truncatus*), and melon‐headed whale (*Pepnocephala electra*). However, descriptions of patterns within these labeled data, and comparisons to literature have not been completed.

Analysis of long‐term acoustic data from these sites can provide new perspectives on temporal presence, particularly for more cryptic species or those averse to human activities. In some cases (e.g., *Kogia* spp.), temporal analyses and documentation of patterns are relatively novel, as few previous studies have had sufficient detections of these species to describe patterns. In the case of rough‐toothed dolphin, description of spatiotemporal trends based on a novel click type described by Ziegenhorn et al. (2022) will represent one of very few descriptions of temporal patterns in this species' behavior. Even in cases where strong patterns in presence have been documented, additional data, particularly data arising from differing methodologies, can prove useful by filling in knowledge gaps. In addition, concurrently analyzing timeseries for all species of odontocetes commonly found in this data set presents an opportunity to compare composition and temporal patterning without concern for differences in sampling and processing regimes.

In this study, timeseries of species' echolocation click detection (henceforth “detections”) were derived from the existing, labeled PAM data set to examine spatiotemporal patterning in the Hawaiian Islands for the eight groupings of odontocetes mentioned above. Composition among sites was evaluated, and significance of temporal patterns was determined using generalized additive models (GAMs) with generalized estimating equations (GEEs). Significant temporal patterns were described and compared to available previous literature. The patterns described improve upon previous research for many species where records at various scales (e.g., lunar, seasonal, and yearly) are lacking, providing a baseline for studies of behavior. The breadth of results presented here highlights the utility of long‐term, fine‐scale detection data in species monitoring efforts.

## 2 METHODS

### 2.1 Data collection

**FIGURE 1:**
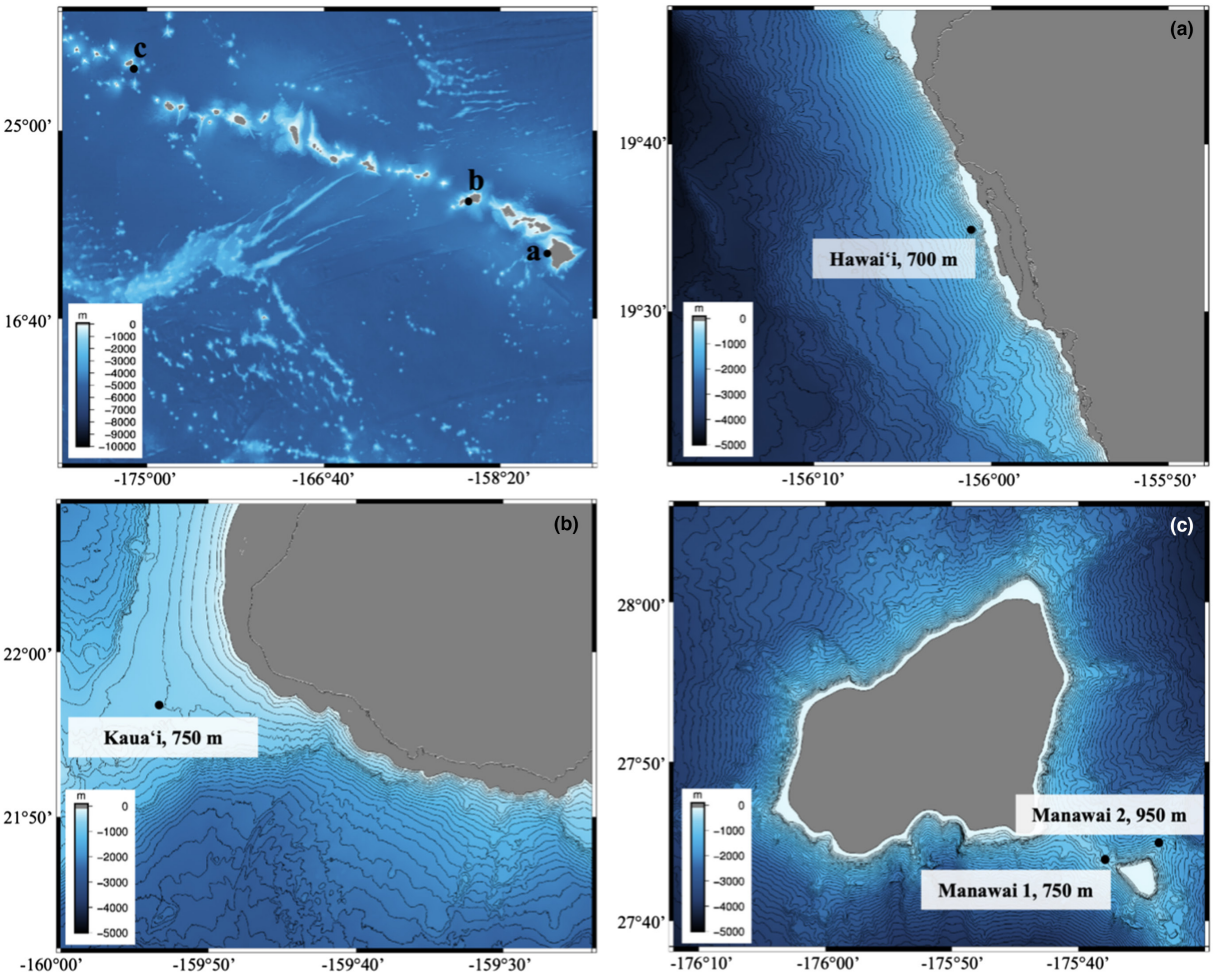
Deployment locations. Recording sites for this study, showing the location and average depth of each site, with 50 m contour lines. Top left panel shows site locations in context of the Hawaiian islands chain. Panels (a–c) show locations of Hawai'i, Kaua'i, and Manawai sites (respectively). Bathymetry data used for the top left panel was accessed via the general bathymetric map of the ocean (GEBCO) 2022 gridded global dataset (accessible here: https://www.gebco.net/data_and_products/gridded_bathymetry_data/#global). Bathymetry data used for panels (a–c) accessed from the Hawai'i mapping research Group at the University of Hawai'i at Manoa (a, b, accessible here: http://www.soest.hawaii.edu/hmrg/multibeam/bathymetry.php) and Pacific Islands ocean observing system (c, accessible here: https://pae‐paha.pacioos.hawaii.edu/thredds/bathymetry.html?dataset=hurl_bathy_60m_nwhi).

Passive acoustic data were collected in the Hawaiian Islands using High Frequency Acoustic Recording Packages (HARPs; Wiggins & Hildebrand, 2007). Deployments used for this study spanned the years 2009–2019 (see Table S1.1). Sites for data collection were off the west coast of Hawai'i Island (henceforth, ‘Hawai'i’), west of Kaua'i, and in the vicinity of Manawai (Figure 1). For the latter location, the exact recording site shifted over the recording period (2009–2017), primarily to combat low‐frequency hydrophone cable strumming from strong currents at depth. As such, two subsites have been designated (henceforth, “Manawai 1,” “Manawai 2”). Deployment setup varied at these sites in terms of recording schedule, instrument depth, and duty cycle regime (i.e., alternating periods of recording and nonrecording; see Table S1.1). Duty cycling was employed to extend battery life and allow for longer deployments. Data from these sites were recorded at a 200 or 320 kHz sampling frequency (16‐bit quantization) at depths ranging from 550 to 1150 m (see Table S1.1). All hydrophones were buoyed approximately 10–30 m from the seafloor, with a maximum detection range of approximately 5 km for most odontocete echolocation clicks (Frasier, 2015; Hildebrand et al., 2015, 2019).

### 2.2 Data processing

An energy‐based detector was run on all data to identify echolocation clicks, which were then evaluated to determine relevant click features (i.e., timing between clicks in a click train, or “inter‐click interval,” spectral shape, and peak frequency; e.g., Buchanan et al., 2021; Lu et al., 2013). These clicks were then clustered using unsupervised clustering methods, resulting in several echolocation click types (e.g., Frasier et al., 2017; Reyes Reyes et al., 2015). These types were identified to species level where possible based on known records and auxiliary data from the region. Then, types were used as classes to train a neural network‐based classifier, which was run on all data to label the entirety of the data set as either one of the echolocation click types or noise (e.g., Frasier, 2021; Ziegenhorn et al., 2022).

Detections of all types were originally binned in 5‐min increments for network training and labelling. Numbers of clicks in 5‐min bins were multiplied by type‐specific precision values (a measurement of the percentage of all network labels of a given type that were true positives) to approximate the number of “true” clicks in that bin. Bins were retained for timeseries only if they had more than a certain number of “true” clicks (>50 for delphinids, >20 for beaked whales and *Kogia* spp., based on clicking rates for these species). Further detail on network precision and accuracy can be found in Ziegenhorn et al. (2022). For false killer whales, neural‐network classifications included many false detections from noise sources, which were removed manually in lieu of this process. Final timeseries were binned in counts of minutes with detections per hour. It was assumed that all minutes within a given 5‐min bin contained clicks of a type if that type was present in the bin. Short‐finned pilot whale and stenellids each had two associated click types, which were left separate during classification to improve network performance but were determined to not represent different species or populations (Ziegenhorn et al., 2022). Timeseries for these types were consolidated to one stenellid type and one short‐finned pilot whale type for this analysis.

As the full data set for all sites included both continuous and duty cycled deployments, it was necessary to further account for the effects of duty cycles (see Table S1.1). This was done on both a site and type‐specific basis, as duty cycling does not necessarily affect all species equally (Stanistreet et al., 2016). For each type at each site, timeseries of detections were subsampled to replicate each duty cycle used at that site. These subsamples were evaluated in comparison to the continuous timeseries to determine what percentage of minutes per hour of detections would have been lost if the given duty cycle had been in effect. This was repeated for all duty cycles at each site. The resulting percentages of missed minutes per hour were used to linearly boost the counts of minutes per hour in duty cycled deployments for each type. Manawai subsites were combined into one “Manawai” site due to their close proximity.

### 2.3 Data analysis

Full timeseries data for each type were plotted along with lunar and solar information extracted using the *suncalc* package in R (Thieurmel & Achraf, 2019). In addition, seasonal variation in detection was calculated as average hours per week of detections across available years of data (Figure 1). Seasons were defined as winter (January through March), spring (April through June), summer (July through September), and fall (October through December). Percentage of days with detections of each type was evaluated by‐site to examine compositional differences between sites. A Bray–Curtis dissimilarity test was used to look at compositional relationships among sites (Bray & Curtis, 1957). This test compares species diversity and abundance at each site to that of a given “focal site,” resulting in a value of one if the same species are present in the same numbers, and zero if the sites have none of the same species. For this study, percentage of recording days with a given species detected was used as a proxy for abundance at that site, similar to (Baumann‐Pickering et al., 2014). Multiple iterations of Bray–Curtis tests were performed, with each site treated as the focal site. It was determined from this that relationships among sites were interpretable regardless of focal site choice. Hawai'i was arbitrarily chosen as the final focal site for analysis, and data were split up by‐season to evaluate how composition changed.

Basic temporal models were built using R (R Core Team, 2021) to test the significance of predictors (hour of day, lunar fraction, and Julian day) for each type at each site. Lunar illumination and phase were extracted using the *suncalc* package in R (R Core Team, 2021; Thieurmel & Achraf, 2019), but were ultimately removed from final models due to a lack of compelling relationships with detections. Year was included only at Hawai'i where more than 5 years of consecutive data were available. To simplify modeling, minutes per hour were transformed into binomial presence–absence (i.e., an hour was given a value of one if clicks were present, and zero otherwise) for use as the response variable. For a given type, models were evaluated only if the number of total detection hours exceeded 100. Temporal autocorrelation was assessed using the residuals of a basic generalized linear model (GLM) including all variables. The time step for temporal blocking was determined as the point where the autocorrelation of residuals dropped below 0.1. These time steps were used to define clusters of self‐similar data in subsequent modeling steps.

Generalized additive models (GAMs) with generalized estimating equations (GEEs) were used as a model framework to additionally combat the autocorrelation expected in continuous passive acoustic data sets (Bailey et al., 2013; Merkens et al., 2019; Pirotta et al., 2011). Lunar illumination was included as a smoothed or linear term depending on Quasilikelihood under the Independence model Criterion (QIC) values (Pan, 2001) for a basic model including only lunar illumination as a predictor. Julian day and hour of day were included as cyclic smooths, and year was included as a smooth at Hawai'i given the long timeseries at this site. All smoothed terms used multivariate splines (*mSpline()*, from the *splines2* package in R; Wang & Yan, 2021) with four knots to avoid overfitting. Variable significance was determined based on *p*‐values calculated via an ANOVA of the final model, with *p* < .05 as the cutoff for significance. Nonsignificant (*p* ≥ .05) terms were removed before creation of the final model. Term order in the final model was determined through backwards selection, based on comparing QICs from models with each term removed sequentially to evaluate their relative contribution to the model's predictive power. *p*‐value, degrees of freedom, and chi‐squared values from an ANOVA of final models were noted.

For final models, partial‐fit plots were developed to visualize the probability of species detection in response to each temporal variable considered. To create these plots, model coefficients were bootstrapped using the covariance matrix included in the model output. Bootstrapped values were then used to determine the spread of possible spline fits; confidence intervals were taken from the 2.5% and 97.5% percentiles of this distribution. Best fit splines were calculated using the model coefficients from final models and plotted along with confidence intervals (code adapted from Pirotta et al., 2011). Effort was plotted across the bottom of these plots and observed patterns in significant predictors were compared to previous literature as well as among sites for included species. Models were evaluated using binned residuals via the *performance* package in R (Lüdecke et al., 2021). This process split data into bins based on fitted values, and plotted average fitted values against average residual values for each bin. The results of this were evaluated based on how many values fell outside of the theoretical 95% error bounds; a good model was expected to have at least 95% of values fall within these error bounds (Gelman & Hill, 2006). Coefficients of discrimination (also known as Tjur's *R* ^2^) were also evaluated for this final model using *r2_tjur()* in the *performance* package in R, as typically used for generalized linear models with binary outcomes (Tjur, 2009).

## 3 RESULTS

### 3.1 Species detection

**FIGURE 2:**
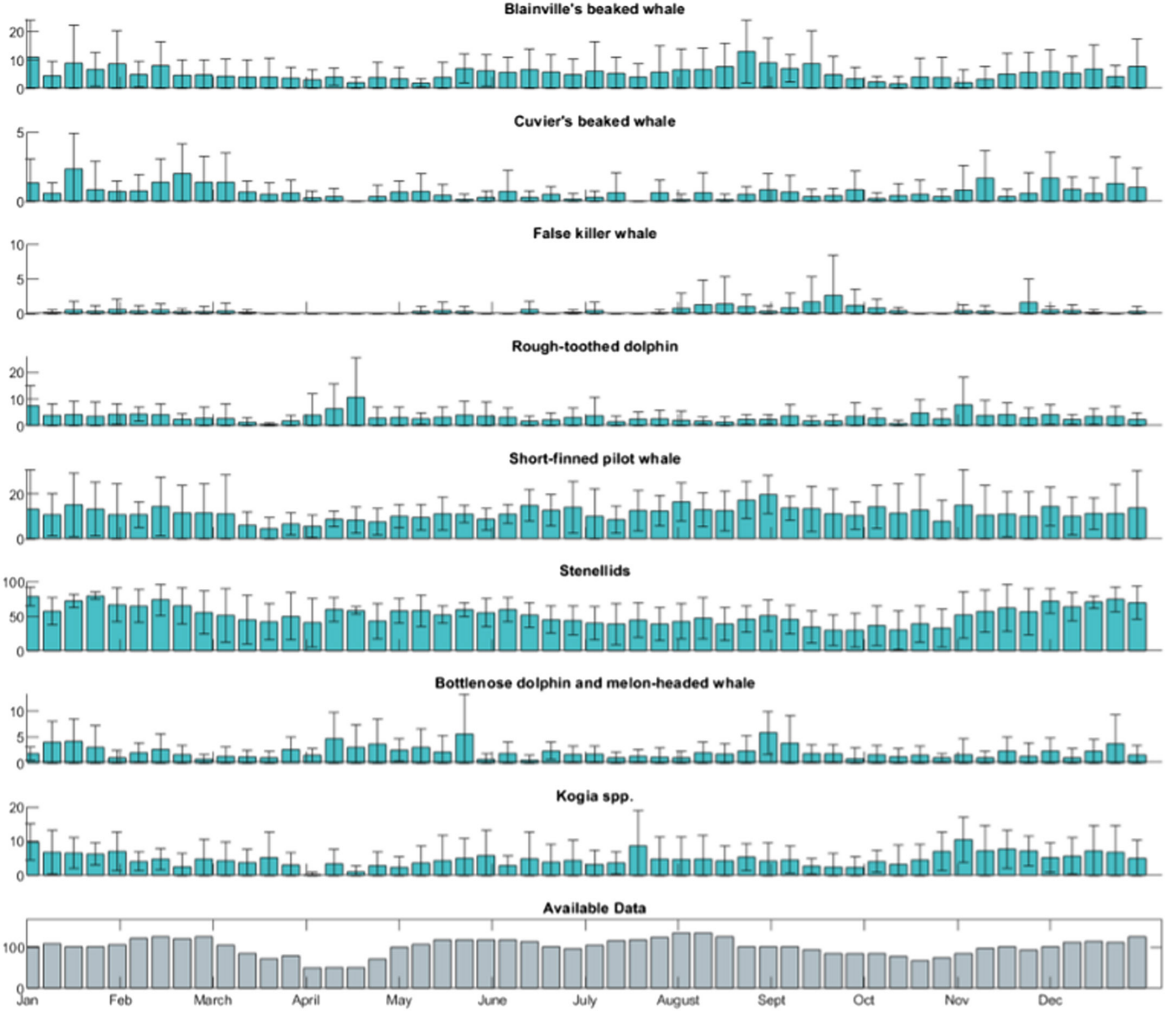
Seasonal detections at Hawai'i. Seasonal detections (hours per week) of all types at Hawai'i. Note that *Y*‐axis scales vary for each species. Average detections with standard error across available years of data is shown. Detections are not corrected for total hours of available data during a given week.

**FIGURE 3:**
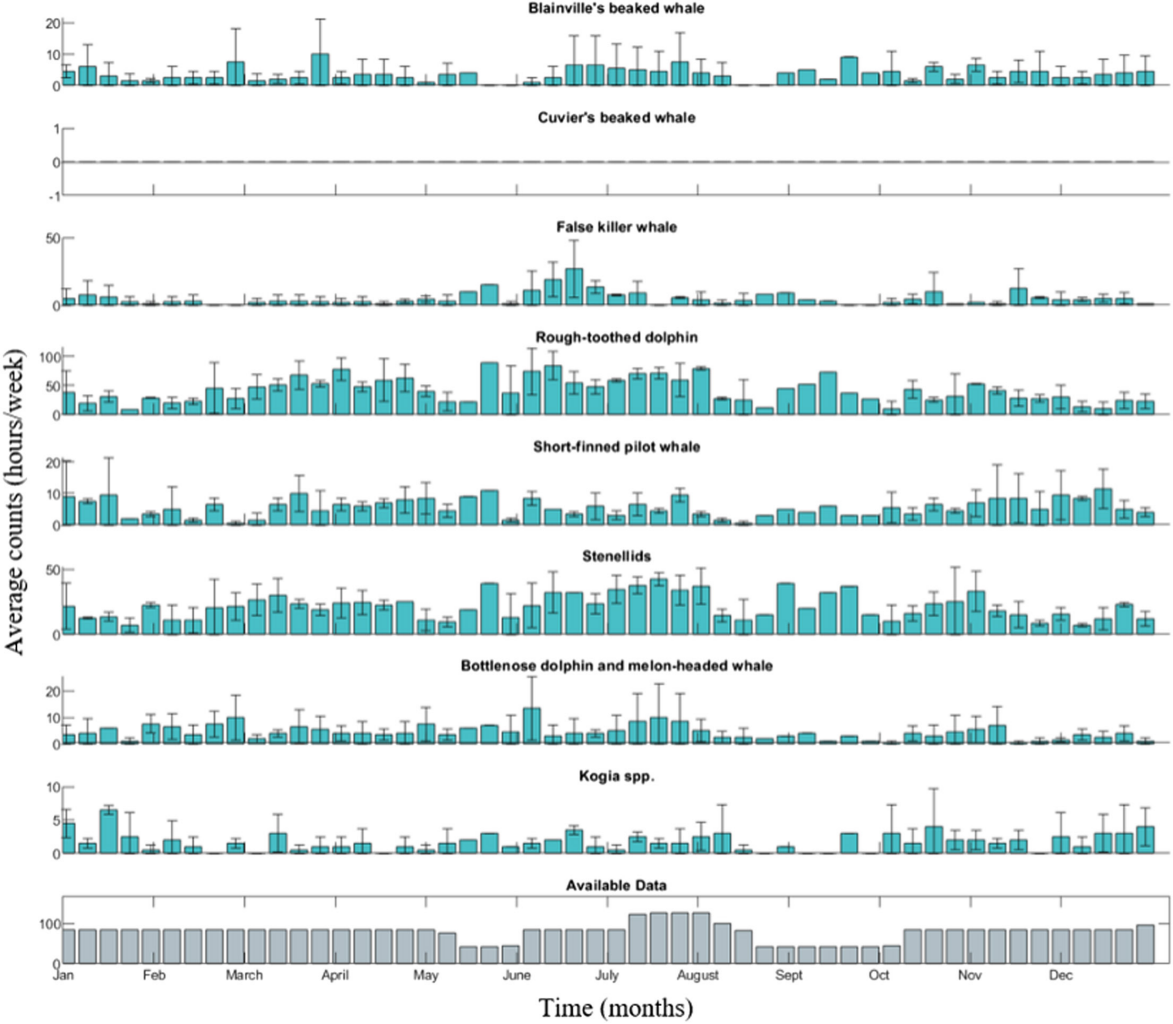
Seasonal detections at Kaua'i. Seasonal detections (hours per week) of all types at Kaua'i. Note that *Y*‐axis scales vary for each species. Average detections with standard error across available years of data is shown. Detections are not corrected for total hours of available data during a given week.

**FIGURE 4:**
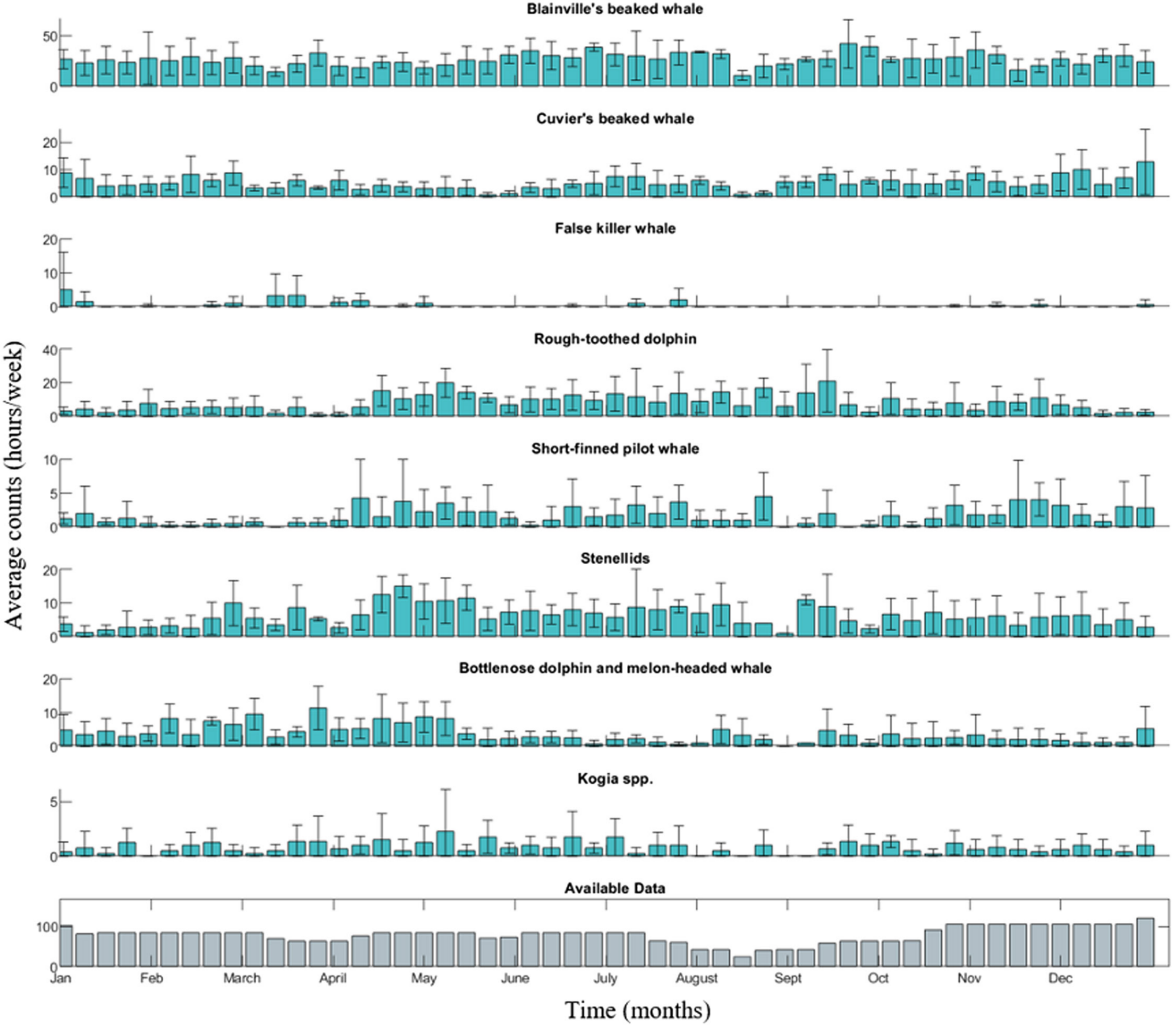
Seasonal detections at Manawai. Seasonal detections (hours per week) of all types at Manawai. Average detections with standard error across available years of data is shown. Detections are not corrected for total hours of available data during a given week.

Species detections varied among types and sites (Figures 2, 3, 4). All types were found at all sites except for Cuvier's beaked whales, which were not found at Kaua'i (Figure 3). There was variable effort across weeks over all years. For Kaua'i, a maximum of three years of data were available for a given week, resulting in large standard errors in many cases.

### 3.2 Species composition

**FIGURE 5:**
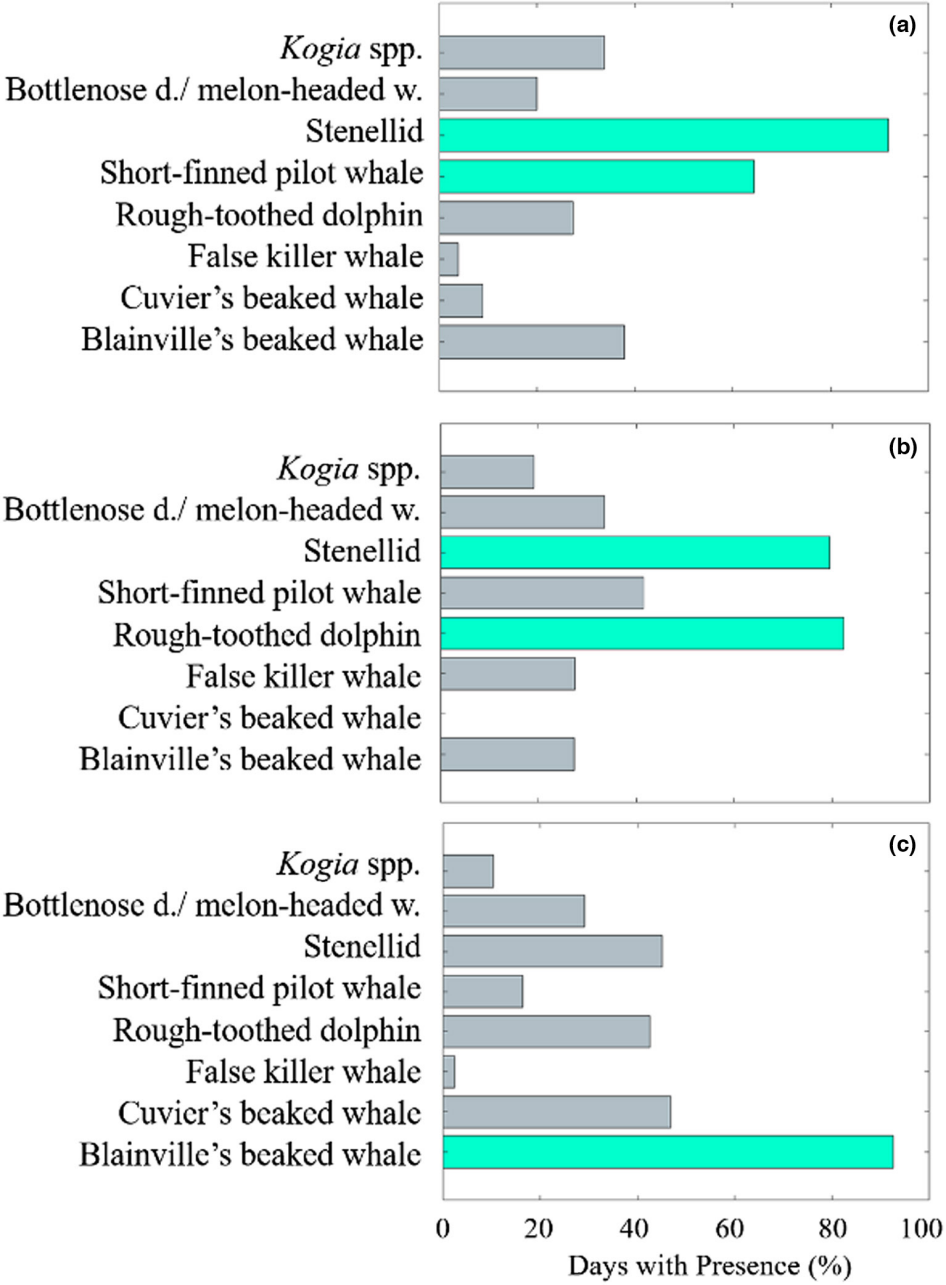
Percentage days with detections. Percent days out of total recording days at each site with detections of each type. Sites are (a) Hawai'i, (b) Kaua'i, and (c) Manawai. Values greater than 50% are shown in green.

Species composition varied by site as well as by season. At Hawai'i, the most common species detected were stenellid dolphins (present 90% of days), short‐finned pilot whales (62%), and Blainville's beaked whales (38%; Figure 5a). Seasonal compositional shifts were limited (<16% in all cases), and the relative commonality of types changed very little (see Figure S2.1). At Kaua'i, stenellids were also commonly detected (79%), along with rough‐toothed dolphins (82%) and then short‐finned pilot whales (40%; Figure 5b). For this site, variability was higher (seasonal differences up to 23%), though again these changes had little effect on the relative commonalities of types. An exception to this was the relationship between false killer whales and Blainville's beaked whales; false killer whales were more commonly detected than Blainville's beaked whales in summer, but not fall (see Figure S2.2). At Manawai, Blainville's beaked whales were by far the most commonly detected species (91%; Figure 5c), though stenellids and Cuvier's beaked whales were also common at this site (58% and 57% of days). This site had the most seasonal variability in presence, with a highest difference of 29% (Table 1, see Figure S2.3). Manawai saw the most notable change from winter to spring, with detections of nearly half the species considered increasing by at least 10% between these seasons (see Figure S2.3). Seasonal shifts in detections were most dramatic for rough‐toothed dolphins (largest change (23%) from winter to spring; Table 1).

**TABLE 1:** Seasonal differences in percent presence

Percent difference		Type							
		False killer whale	Rough‐toothed dolphin	Short‐finned pilot whale	Bottlenose d./melon‐headed w.	Blainville's beaked whale	Cuvier's beaked whale	Stenellids	*Kogia* spp.
Hawai'i	SpSu	1.52	−5.76	8.82	−5.06	7.98	−2.19	−5.73	2.47
	SuFa	2.82	7.81	**−10.9**	−0.12	−9.58	3.95	−8.16	7.28
	FaWi	−2.94	3.33	7.25	3.36	**10.3**	4.57	**16.8**	1.36
	WiSp	−1.40	−5.38	−5.19	1.82	−8.74	−6.32	−2.90	**−11.1**
Kaua'i	SpSu	7.99	**−10.4**	−9.32	1.37	−7.97	NA	−6.20	3.66
	SuFa	**−12.7**	−4.02	8.38	−9.37	**18.4**	NA	−2.92	1.05
	FaWi	3.08	−9.03	−2.85	5.38	−9.34	NA	−8.77	2.36
	WiSp	1.67	**23.4**	3.79	2.98	−1.06	NA	**17.9**	−7.07
Manawai	SpSu	−2.13	3.43	2.30	**−23.9**	3.30	3.98	−6.62	−2.01
	SuFa	−0.63	**−14.5**	−0.16	1.89	−0.51	9.09	−7.13	−1.24
	FaWi	1.63	**−12.3**	−7.90	6.58	−1.29	0.82	**−15.2**	−0.03
	WiSp	1.13	**23.4**	5.76	**15.4**	−1.49	**−13.9**	**29.0**	3.28

*Note*: Seasonal changes in percentage of recording days with presence at all sites. Season codes are SpSu, spring to summer change; SuFa, summer to fall; FaWi, fall to winter; WiSp, winter to spring. Changes greater than ± 10 are given in bold.

**FIGURE 6:**
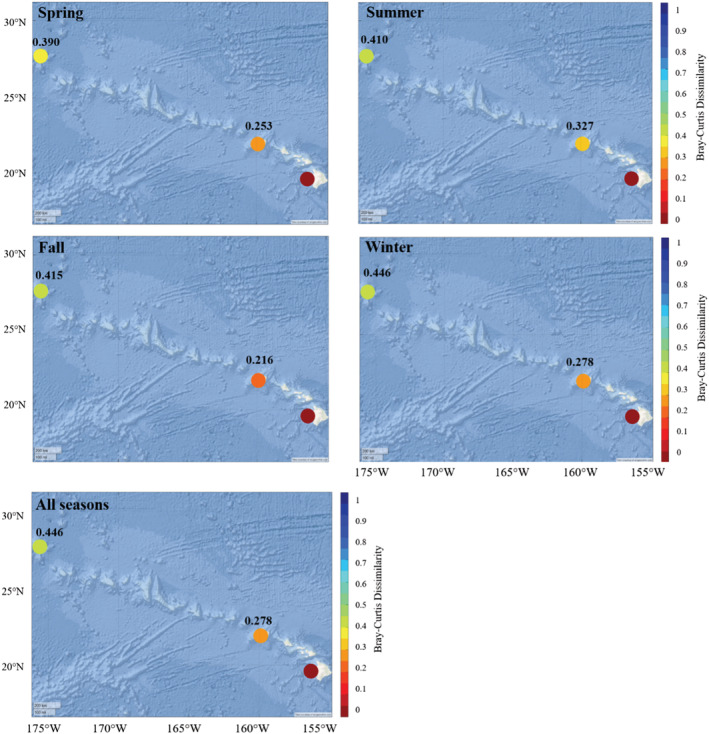
Bray–Curtis dissimilarity map. Depiction of Bray–Curtis dissimilarity among sites with Hawai'i as the focal site in each season, as well as overall. Color is used to depict Bray–Curtis value between the focal site and each other site, with red representing sites where composition is the same, and blue representing sites with no overlap in species. Exact Bray–Curtis values are shown above corresponding sites. Basemap image is the intellectual property of Esri and is used herein with permission. Copyright © 2022 Esri and its licensors. All rights reserved.

Bray–Curtis dissimilarity tests further quantified site‐specific compositional differences. With Hawai'i as the focal site, Kaua'i was most similar, with composition at Manawai being less similar (Figure 6). Smaller compositional shifts across seasons at Hawai'i and Kaua'i were reflected by lower Bray–Curtis values (Figure 6). Composition among all sites was most similar in the spring, and most different in the winter, though composition between Hawai'i and Kaua'i was most similar in the fall (Figure 6).

### 3.3 Temporal patterns

Temporal patterns were modeled with GAM‐GEEs using hourly presence or absence of all types as response variable and hour of day, Julian day (as a proxy for seasonal changes), and year (Hawai'i only) as explanatory variables. This provided information on the significance of these temporal scales to detection of species or types (Table 2). Reliable patterns were not discernible for types with fewer than 100 h of detection at a site. As such, these types were not considered in modeling and pattern description efforts. Across all species and sites, hour of day (local time) was the most common significant variable, with only three cases in which it was not significant (*p* > .05; Table 2). Julian day was significant for all but five models. At Hawai'i, where year was considered as a smoothed variable, year was a significant predictor for all species (*p* < .05 in all cases).

**TABLE 2:** Model results

Model results (sig, df, χ^2^)		Type							
		False killer whale	Rough‐toothed dolphin	Short‐finned pilot whale	Bottlenose d./melon‐headed w.	Blainville's beaked whale	Cuvier's beaked whale	Stenellids	*Kogia* spp.
Hawai'i	Hour		*p* < .001, 5, 232	*p* < .001, 2, 294	*p* < .001, 2, 373	*p* < .01, 2, 9.93		*p* < .001, 2, 6478	*p* < .001, 2, 33.2
	Julian day	*p* < .01, 2, 12.3	*p* < .001, 2, 34.3				*p* < .001, 2, 38.4	*p* < .001, 2, 469	*p* < .01, 2, 10.3
	Year	*p* < .001, 5, 34.8	*p* < .001, 2, 197	*p* < .001, 5, 74.5	*p* < .001, 5, 74.5	*p* < .001, 5, 48.2	*p* < .001, 5, 43.0	*p* < .001, 5, 386	*p* < .001, 5, 58.5
	nPres	141	1036	3843	678	1836	234	17,509	1612
Kaua'i	Hour	*p* < .01, 2, 10.8	*p* < .001, 2, 311	*p* < .001, 2, 64.4	*p* < .001, 2, 309	*p* < .001, 2, 17.2	—	*p* < .001, 2, 233	
	Julian day	*p* < .05, 2, 6.89	*p* < .001, 2, 34.1	*p* < .01, 2, 10.1	*p* < .001, 2, 16.1		—	*p* < .001, 2, 21.8	*p* < .05, 2, 8.68
	nPres	480	4038	546	436	346	<100	2049	170
Manawai	Hour	—	*p* < .001, 2, 337	*p* < .001, 2, 126	*p* < .001, 2, 357	*p* < .01, 2, 10.2	*p* < .001, 2, 46.3	*p* < .001, 2, 481	*p* < .05, 2, 7.70
	Julian day	—	*p* < .001, 2, 86.6		*p* < .001, 2, 62.9	*p* < .001, 2, 17.9	*p* < .001, 2, 48.8	*p* < .001, 2, 53.8	*p* < .05, 2, 8.63
	nPres	<100	1558	358	1233	5343	1059	745	162

*Note*: Table showing model results for all types at all sites. Included variables are hour of day (hour), percentage of lunar illumination (lunar), Julian day, and year. Number of hours with presence of a type is given by nPres. Significant variables (sig) are marked with *p* < .001, *p* < .01, or *p* < .05. Degrees of freedom (d) and chi‐squared values (*χ* ^2^) are given for all variables. Blank spaces indicate lack of significant variables. Gray boxes indicate insufficient presence hours for modeling.

Performance of models was variable, with several models (5 of 22) reaching >90% of values within the error bounds of the binned residuals plot, though no model reached >95% (Table 3). These values did not necessarily correspond to higher *R* ^2^ values, nor was there a relationship between more detection hours and better model performance. Model results are discussed in further detail below. Full timeseries of detections for each species and site can be found in Figures S3.1–S3.23.

**TABLE 3:** Model evaluation

Type	Hawai'i			Kaua'i			Manawai		
	nPres	Percent res	Res *R* ^2^	nPres	Percent res	Res *R* ^2^	nPres	Percent res	Res *R* ^2^
False killer whale	141	22	.005	480	84	.005	<100	—	—
Rough‐toothed dolphin	1036	75	.020	4038	**91**	.067	1558	87	.034
Short‐finned pilot whale	3843	82	.028	546	86	.006	358	78	.005
Bottlenose d./Melon‐headed w.	678	59	.015	436	60	.021	745	64	.026
Blainville's beaked whale	1836	73	.009	346	**91**	.001	5343	**96**	.003
Cuvier's beaked whale	234	48	.002	<100	—	—	1095	**92**	.004
Stenellids	17,509	47	.322	2049	85	.033	1233	87	.026
*Kogia* spp.	1612	86	.006	170	68	.001	162	59	.006

*Note*: Table showing model evaluation for all types at all sites with number of hours with presence (nPres), percentage of values that were within 95% error bounds in binned residual plots (% res), and Tjur's *R* ^2^ values for each model (res *R* ^2^). Gray boxes indicate insufficient presence hours for modeling. Models with greater than 90% percent res values are bolded.

#### 3.3.1 Diel patterns

**FIGURE 7:**
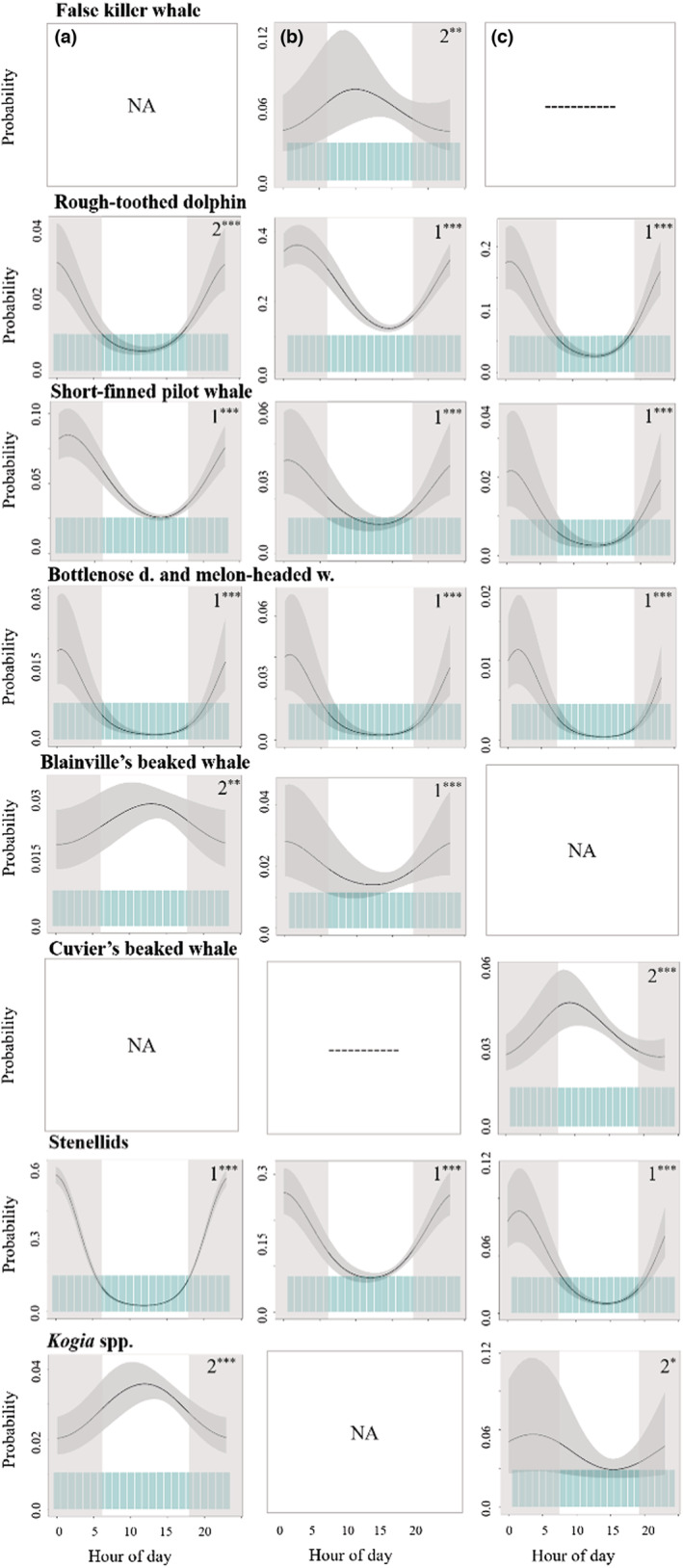
Diel patterns. Partial‐fit plots for hour of day (in HST) for all types at (a) Hawai'i, (b) Kaua'i, (c) Manawai. Plots show the probability of species detections with 2.5 and 97.5 percentile value confidence intervals. Blue polygons at the bottom of each plot give the distribution of the underlying data. Model order is given for significant variables (1st–4th). Asterisks show significance level (*** = *p* < .005, ** = *p* < .01, * = *p* < .05). Nonsignificance is denoted by ‘NA’; sites without a model are represented by ‘—’.

Clear diel patterns were found for most species and sites (Figure 7). For false killer whales, Cuvier's beaked whales, and *Kogia* spp., detections increased during daylight hours. Probability of false killer whale detection was higher during mid‐morning at Kaua'i (peak at 10:00–11:00 HST; Figure 7). For Cuvier's beaked whales, hour of day was a significant driver at Manawai with a peak in detections at 8:00–9:00. For this species at the Hawai'i site, timeseries examination revealed that diel detection changed over the years considered. Little to no diel pattern was present during the 2010 peak in detections, but a distinct increase in detection during daylight hours was seen in later years. Peak detection for *Kogia* spp. was slightly later in the day (around 13:00), with a strong diel trend only at Hawai'i. For rough‐toothed dolphins, the bottlenose dolphin and melon‐headed whale class, short‐finned pilot whales, and stenellids, models showed a distinct diel trend, with echolocation detections being much lower in daylight hours (Figure 7). For stenellids, this was particularly strong at Hawai'i (Figure 7). For rough‐toothed dolphins, there were fewer detections during midday hours at Hawai'i and Manawai, but this dip occurred slightly later (14:00–15:00 HST) for Kaua'i. For short‐finned pilot whales, diel patterning varied over considered years at Hawai'i (see Figure S3.16). Diel patterning was also more variable for Blainville's beaked whales. For this species, there was an overall decline in detection throughout daylight hours at Kaua'i. However, at Hawai'i there was a slight increase in detections during midday. Examination of timeseries at this site showed that this diel patterning was much less evident during time periods with higher detections (e.g., 2011, 2017, see Figure S3.22–S3.23).

#### 3.3.2 Seasonal patterns

**FIGURE 8:**
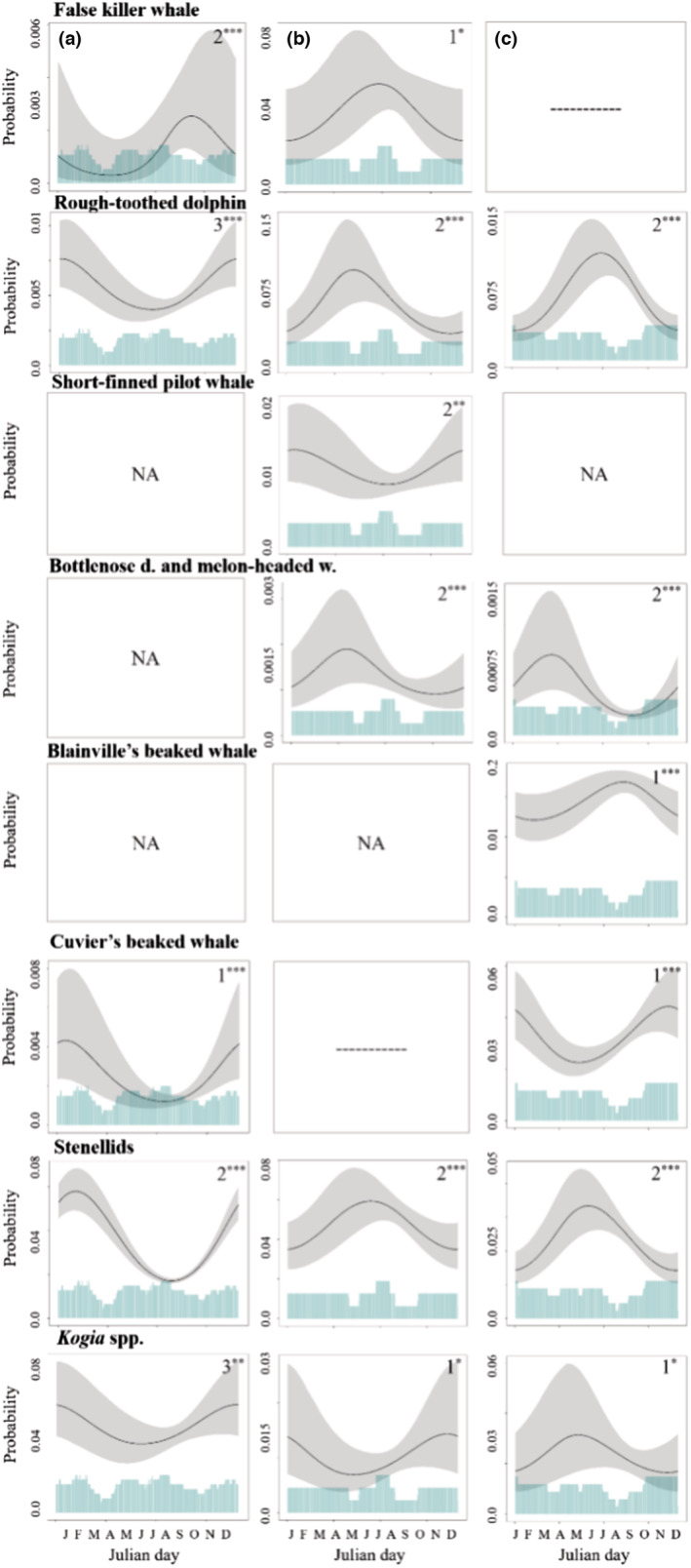
Seasonal patterns. Partial‐fit plots for Julian day for all types at (a) Hawai'i, (b) Kaua'i, (c) Manawai. Plots show the probability of species detections with 2.5 and 97.5 percentile value confidence intervals. Blue polygons at the bottom of each plot give the distribution of the underlying data. Model order is given for significant variables (1st–4th). Asterisks show significance level (*** = *p* < .005, ** = *p* < .01, * = *p* < .05). The first letter of each month is shown instead of day of year, for ease of readability. Nonsignificance is denoted by ‘NA’; sites without a model are represented by ‘—’.

Seasonal patterning varied among species and sites. For false killer whales, detections were more common in the fall at Hawai'i, versus an earlier peak in summer at Kaua'i (Figure 8). For rough‐toothed dolphins, detections were more frequent in the spring at Kaua'i and summer at Manawai, but in the winter for Hawai'i (Figure 8). Short‐finned pilot whales had a seasonal pattern only at Kaua'i, with higher detections during winter. For stenellids, detections were more common in spring at Kaua'i and Manawai. At Hawai'i, stenellids were detected in the highest numbers during winter (Figure 8). Seasonal patterning for Blainville's beaked whales was only apparent at Manawai, where there was a slight increase in detections in July–August. Patterning for Cuvier's beaked whales indicated a fall–winter peak in detections at both Manawai as well as Hawai'i. For the bottlenose dolphins and melon‐headed whale class, seasonal trends were significant at Kaua'i and Manawai, with a slight peak in detections during the late winter and early spring at both sites.

#### 3.3.3 Multi‐year patterns

**FIGURE 9:**
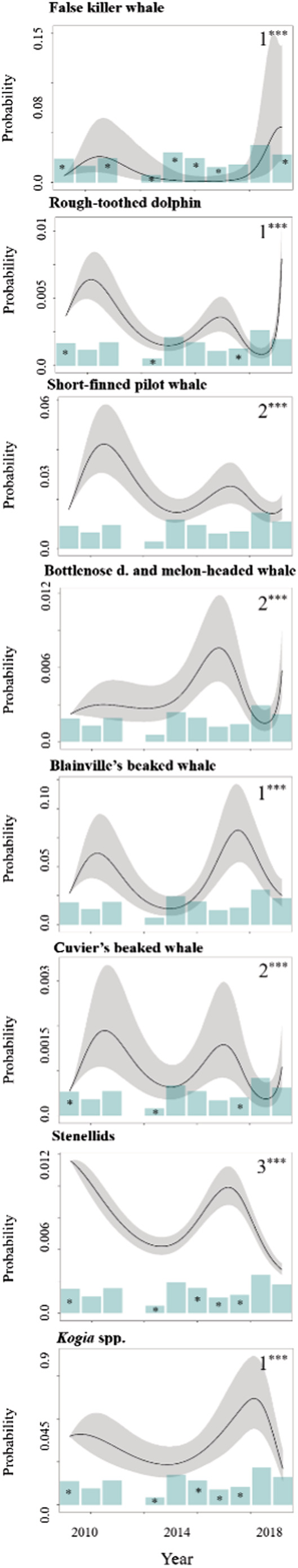
Multi‐year patterns. Partial‐fit plots for year for all types at Hawai'i. Plots show the probability of species detections with 2.5 and 97.5 percentile value confidence intervals. Blue polygons at the bottom of each plot give the distribution of the underlying data. Model order is given for significant variables (1st–4th). Asterisks show significance level (*** = *p* < .005, ** = *p* < .01, * = *p* < .05). The first letter of each month is shown instead of day of year, for ease of readability. Asterisks on histogram data indicate years in which data from a key season for this type was missing.

Long‐term trends of species presence at Hawai'i were significant for all types considered (Figure 9). Short‐finned pilot whales, rough‐toothed dolphins, Blainville's beaked whales, and Cuvier's beaked whales all had markedly similar patterns in the years considered. For these species, predicted presence was highest in 2010–2011 and 2016 and lowest in 2014 and 2018 (Figure 9). These species (with the exception of short‐finned pilot whales) then saw an increase in presence in 2019 (Figure 9). For rough‐toothed dolphins and Cuvier's beaked whales, years with lower probability of presence corresponded partially to years in which key seasons for those species were times of no effort (Figure 9). However, this did not hold true for the other species with this pattern. Variations on this pattern were observed for stenellids as well as the bottlenose dolphin and melon‐headed whale class. For stenellids, a decrease was observed until 2014 and a 2016 peak was present before a decline again until the end of the data set. The bottlenose dolphin and melon‐headed whale class had a 2016 peak and 2018 decrease similar to many other species, but fairly consistent predicted presence prior to 2016. False killer whales had a peak in 2010–2011 similar to what was seen for other species, but then consistently low presence after this (large error bars make it difficult to conclude much about the trend post‐2017). *Kogia* spp. had a slight dip in 2014, but a shifted peak with highest predicted presence in 2017–2018 before a steep 2019 drop off (Figure 9).

## 4 DISCUSSION

This study analyzed timeseries of species echolocation click detection for 8+ species of odontocetes in the Hawaiian Islands region at multiple sites and over a wide range of years and seasons. This allowed for novel comparisons of species composition and commonality among sites.

Composition among sites generally followed expected trends, with observed Bray–Curtis values being presumably mostly related to spatial proximity. Examining changes in these relationships by‐season demonstrated the robustness of the overall compositional results. Despite shifts in species detections among season that were demonstrated both in by‐season compositional results as well as through temporal modeling, seasonal Bray–Curtis relationships remained markedly stable and similar to the full data result (Figure 6). While relationships between sites did not differ much, the spread of Bray–Curtis values compared to Hawai'i did change, with sites being most similar in the spring and least similar in the winter. This is an interesting result that is mostly driven by changes in composition at Manawai. In the spring, higher similarity among sites is driven primarily by the increase in stenellids making composition at Manawai more like Hawai'i than in other seasons. In the fall, lower similarity compared to Hawai'i is driven by fewer stenellids and more Cuvier's beaked whales at Manawai. The degree of seasonal changes in species detections (e.g., most consistent at Hawai'i, least consistent at Manawai) may be driven by patterns in underlying oceanographic features, particularly those that affect prey availability (e.g., fronts, chlorophyll‐a concentration). In particular, the higher degree of seasonal composition shifts at Manawai is likely related to movements of the Transition Zone Chlorophyll Front, which is closer to the islands in the winter and enhances local productivity (Polovina et al., 2017). Differences in productivity between the Main Hawaiian Islands and Northwestern Hawaiian Islands are complex, and this likely leads to variations in species behavior. Such differences are the subject of ongoing research efforts and will be the focus of a future paper.

Some distributional differences observed were also well supported by existing sighting and tag records. The lack of Cuvier's beaked whales at Kaua'i and higher presence at both Hawai'i and Manawai is corroborated by existing sighting records (Baird, 2016). Rough‐toothed dolphin sighting rates are much higher off Kaua'i than off Hawai'i (Baird, Webster, Mahaffy, et al., 2008), similar to our results. Documented trends in *Kogia* spp. (specifically *K. sima*) sightings also match our findings, in which detections were more common off Hawai'i than off Kaua'i (Baird et al., 2021). Other compositional differences noted in this study may be similarly validated by future tagging and visual survey efforts around the islands.

Using a neural network classifier, rather than manual labeling, to derive timeseries of detections necessarily resulted in some classification error. In this study, we used type and site‐specific precision values to help mitigate these errors and minimize the obfuscation of actual patterns. The false killer whale class presents an opportunity to explore this in some detail. Due to low precision values (many misclassifications with noise), manual review was completed for all false killer whale detections, and this manually modified data set was used moving forward (Ziegenhorn et al., 2022). When comparing this timeseries to the precision‐modified timeseries, we might have used otherwise, it is clear that noise detections would have created artificial patterns. As an example, confusion with boat noise results in a diel pattern (higher detections during daylight) in the precision‐based data set at Hawai'i (see Figure S3.24). Such effects are less pronounced at Kaua'i and Manawai. This level of misclassification is more egregious than was seen for other sites and species, where the precision‐based reductions were likely enough to mitigate errors. However, it bears acknowledging that there may be residual errors in the patterns observed. Residual classification errors would likely affect short‐finned pilot whales at Manawai and the bottlenose dolphin and melon‐headed whale class at Hawai'i more than other types, based on established precision values. Further study using this data set might consider evaluating whether patterning existed in misclassifications and how that might be used to better account for error in resulting timeseries.

For short‐finned pilot whales as well as stenellids, multiple neural network classes existed for each type; these were combined for this analysis. Differences in stenellid click types were due to artificial spectral peaks introduced by a 25 kHz crossover frequency between low and high frequency hydrophones in some HARP setups (see Table S1.1). However, for short‐finned pilot whales, it is possible that the types we combined could have different patterns in the data. In this case, reasoning for combining types was partially due to their scarcity, particularly at Manawai. If short‐finned pilot whale classes had been left separate, one or both types might have had insufficient data for modeling at this site.

One aim of this study was to further examine temporal patterning to determine the species makeup of the stenellid and combined bottlenose dolphin and melon‐headed whale types. For stenellids, the diel trends observed point to potentially different makeups of the types among sites. In addition to this, the preference of striped dolphins for deeper water (Baird, Webster, et al., 2013) and apparent lack of resident population of spotted dolphins near Kaua'i (Baird & Webster, 2020) may suggest that, at least at this site, the stenellid type is primarily composed of spinner dolphins. Site‐specific differences in type makeup may also hold true for the bottlenose dolphin and melon‐headed whale type. Some of the results presented (i.e., strong diel cycle) may suggest that melon‐headed whales make up a significant portion of this type. However, detailed sighting records from the Main Hawaiian Islands indicate a much higher presence of bottlenose dolphins near Hawai'i and Kaua'i (Baird, Webster, et al., 2013). Based on this information, it is likely that the type makeup in the Main Hawaiian Islands is mostly bottlenose dolphins, though this may be different at Manawai. Discrimination of these species within this type might be possible with the addition of examining characteristics of underlying whistles that often accompany clicks of these types.

Differences in cue rate (i.e., the rate at which vocalizations are produced by an individual) among species may have led to some variation in detectability in this data set. However, considering acoustic presence on a relatively coarse scale compared to the scale of cue rate variability should have mitigated such issues. Species‐specific thresholds for “true” detections also served to minimize differences caused by cue rate. Additionally, it is worth noting that the species considered produce a variety of other vocalizations that were not included in this study. Incorporation of these vocalizations (e.g., whistles, buzzes, and burst pulses) would further contribute to fully describing acoustic presence patterns of the species considered.

There are likely some vocalizations from pygmy killer whales, *Feresa attenuata*, in our data set due to a resident population off the coast of Hawai'i (McSweeney et al., 2009). These vocalizations would bolster detections of some species at this site, particularly during nighttime hours when this population is thought to forage (Baird, 2018). It is not possible to quantify this effect, though based on knowledge of this species' echolocation (Madsen et al., 2004), it is most likely being misclassified as a fellow delphinid species rather than a beaked whale or *Kogia* spp. The effect of these misclassifications is probably mitigated by the small size of this population (McSweeney et al., 2009), although individuals from this population do use slope waters spanning the depth range of the HARP (Baird et al., 2011). Risso's dolphins, *Grampus griseus*, Fraser's dolphin, *Lagenodelphis hosei*, Longman's beaked whales, *Indopacetus pacificus*, and killer whales, *Orcinus orca*, are also known to be present around the islands but likely represent a very small proportion of misclassifications due to low sighting rates near the HARP sites (Baird, Webster, et al., 2013). Some detections of beaked whales may also be misclassifications of an unidentified beaked whale species first detected at Cross Seamount, Hawai'i; this would be most likely at Kaua'i where this species has been acoustically recorded on the U.S. Pacific Missile Range Facility (PMRF; Manzano‐Roth et al., 2022).

### 4.1 Temporal modeling

Overall, modeled patterns of detections corroborated patterns that were seen in the timeseries. Examining the importance of temporal variables in final models and across species and sites also provided a useful framework for considering temporal patterns of odontocete echolocation click detections in a given region. Hour of day was found to be the most significant temporal driver of species detections in this region, across all species and sites (except Hawai'i, where year was significant for all species; Table 1). Evaluation of models using binned residuals and Tjur's *R* ^2^ suggested that many of the models produced are not particularly good predictors of animal detections (Table 2). This is not an unexpected result, as only temporal patterning was considered here. Models would likely be improved by the inclusion of a variety of environmental variables that have been shown to be correlated with presence of odontocetes (e.g., chlorophyll‐a, salinity, and temperature). As the purpose of this study was to highlight spatiotemporal patterning rather than provide predictive models, the poor fit of model residuals found here was not of particular concern.

#### 4.1.1 Diel patterns

Hour of day was a significant driver of species detections in nearly all cases. Diel trends for false killer whales corroborated previous acoustic study of the species, which has suggested that the main Hawaiian Islands stock forages primarily during the day (Simonis, 2018). This pattern could also be the result of inshore‐offshore diel movement patterns resulting in animals being nearer to HARP locations (i.e., inshore) during the day, and further away at night. For rough‐toothed dolphins and short‐finned pilot whales, diel patterns across all sites (i.e., less acoustic activity during daylight) match with previous studies of these species in Hawai'i (Owen et al., 2019; Shaff & Baird, 2021; Simonis, 2018). Increased acoustic activity during the night was also observed for the bottlenose dolphin and melon‐headed whale class, which matches with existing knowledge of melon‐headed whale behavior (Baumann‐Pickering et al., 2015; West et al., 2018). Bottlenose dolphins, in contrast, have been known to forage during both day and nighttime periods (Baird, 2016). However, it is possible that the diel pattern seen could be related to spatial movements of a species that is foraging during both time periods.

For Blainville's beaked whales, detections were more frequent during the day at Hawai'i. The daytime peak in detections for this species is congruent with previous studies in Hawai'i which used a subset of this data set (Baumann‐Pickering et al., 2014). However, other regional work on beaked whales has not noted diel differences in foraging‐related behavior (Baird, 2019; Baird, Webster, Schorr, et al., 2008). Fewer detections during the night at Hawai'i may be related to horizontal movements—whales may be moving further offshore, leading to a dip in detections at the HARP site during those hours. Temporal patterning of species off Kaua'i, particularly beaked whales, may also be impacted by frequent sonar events. The Kaua'i HARP is close to the southernmost extent of the PMRF. Previous study on this range has noted that diel behavior of Blainville's beaked whales is impacted by multi‐day Navy training events, with no diel pattern before training events, but peaks in acoustic detections in both morning and afternoon during and after training events (Henderson et al., 2016). More recent research has found that both multiday training events and the presence of mid‐frequency active sonar can greatly reduce the presence of vocalizing whales (Jacobson et al., 2022).

For stenellid dolphins, detections were far fewer during daylight hours, though the degree of this pattern varied among sites. This trend may elucidate species makeup of this type. Spinner dolphins in Hawai'i echolocate primarily at night and spend days resting in shallow bays (Au et al., 2013; Norris, 1995), whereas spotted dolphins echolocate during the day, although still much more actively at night (Baird, 2016; Baird et al., 2001). For *Kogia* species, no regional comparable studies existed. However, a study of *Kogia* spp. temporal behavior from the Gulf of Mexico found higher acoustic presence during the day at some sites, which is consistent with the pattern seen here for Hawai'i (Hildebrand et al., 2019).

#### 4.1.2 Seasonal patterns

For false killer whales and rough‐toothed dolphins, the seasonality of use seen at various sites (Hawai'i and Kaua'i for false killer whales, all modeling sites for rough‐toothed dolphins) have been noted in very few previous studies. Research on the Main Hawaiian Islands insular stock of false killer whales found some spatial variability in use of the Main Hawaiian Islands region for one social cluster of animals, with highest presence west of Hawai'i during spring and early summer (May through July) and west of Kaua'i in summer and early fall (August through October; Baird et al., 2019). This is somewhat in agreement with our results (i.e., spring–summer peak in Kaua'i, Figure S2.2), though peak season in our data from Hawai'i is fall rather than spring (Figure S2.1, Figure 8). As there are few detections of this species in our record at Hawai'i, and previous seasonal variation was noted for only one social cluster so far, more data would be needed to examine the reasons for this discrepancy. The area near the Hawai'i HARP is not a high‐use area for this stock, so this record may not represent their overall use of the Hawai'i Island lee particularly well. It is also worth noting that the Main Hawaiian Islands insular stock is one of three stocks of false killer whales present in the Hawaiian Islands (Bradford et al., 2015), all three of which use the waters around Kaua'i. For two of these three stocks (i.e., Northwestern Hawaiian Islands and Hawai'i pelagic stocks), seasonality of presence has not been documented. Shifting presence of these stocks may also have effects on overall false killer whale detections at Kaua'i (Baird, Oleson, et al., 2013).

Rough‐toothed dolphin detections were higher in the spring–summer in our data from Kaua'i, which matches with previously modeled high‐use areas during these seasons (Pittman et al., 2016). For short‐finned pilot whales, previous work near Kaua'i found seasonality in detections southwest of the island, in a similar location to the HARP (Au et al., 2014). This study also found an increase in detections in the April–June period. Additional study of this species has suggested that animals may spend more time diving further from shore during fall and winter (Owen et al., 2019). In this case, we might expect to see a decrease in detections during these seasons, which matches the general seasonal trend seen at Kaua'i. The lack of seasonality observed at Hawai'i is in accordance with previous studies that have modeled the region west of Hawai'i as high‐use area for this species year round (Pittman et al., 2016).

For Blainville's beaked whales increased detections in the fall at Kaua'i and summer at Hawai'i have been noted in a previous study that used a subset of this HARP data set (Baumann‐Pickering et al., 2014). The patterns shown here match this prior report, though with this larger sample size, the trend was no longer statistically significant. Seasonal patterning here may suggest movement around the island or offshore, potentially related to shifts in a nearby foraging hotspot (Abecassis et al., 2015). Records of Blainville's beaked whale acoustic detections in the PMRF range have noted decreased detections after multiday naval training events (i.e., prolonged sonar activity periods), which occur in February and August (Henderson et al., 2016). This behavior does not seem to have been captured in our data, despite the Kaua'i site location's proximity to the southern edge of the PMRF. Previous research on Cuvier's beaked whales near Hawai'i Island did note a seasonal peak in the fall similar to what was noted here (Baumann‐Pickering et al., 2014). However, other studies of Cuvier's beaked whales in the region have noted that the species uses the waters near Hawai'i Island yearround and saw no seasonal changes (McSweeney et al., 2007). For stenellid dolphins, observed seasonal patterns in presence have not previously been noted. Seasonal trends in *Kogia* spp. detections have no comparable previous records, though recent study of dwarf sperm whales has revealed the presence of a resident population off of Hawai'i, with much lower encounter rates off Kaua'i, particularly on the western side of the island (Baird et al., 2021). No previous studies have considered seasonality at Manawai.

#### 4.1.3 Multiyear patterns

Multiyear analysis at Hawai'i yielded interesting results in several cases, though in most cases these patterns had no comparable records in the literature. A distinct peak in presence in 2010 was observed for rough‐toothed dolphins, short‐finned pilot whales, and Blainville's and Cuvier's beaked whales. In 2014, a dip in presence was observed for many types. Presence then peaked again in 2016 for all types except false killer whale and *Kogia* spp., before a dip in presence in 2018. This decline was particularly sharp for rough‐toothed dolphins, the bottlenose dolphin and melon‐headed whale class, and Cuvier's beaked whale. Some of these dips may be exacerbated by low effort during key seasons (i.e., lower presence for false killer whales in 2014–2016 may be related to lack of recordings during fall in those years). One downside of including year as a smoothed term in this model is that the representative figures necessarily indicate relationships during years where there is little to no data (i.e., 2012, 2013). It is not possible to say whether the true relationship during those years would be similar to what the model suggests, so we refrained from drawing conclusions related to those time periods. Changes in presence may also be linked to oceanographic variables, particularly those that affect the presence of various prey species. The dip in presence in 2018 is particularly interesting as there was continuous effort throughout that year. Future work on this data set using environmental correlates may illuminate the reasons for this decrease in detections.

## 5 CONCLUSIONS

Discussion of various temporal predictors in relation to previous studies highlights the importance of continued monitoring efforts, in this region and others, to inform understanding of odontocete spatiotemporal patterns. This is especially true for species where this study presents the first regional description of a given pattern, or previous records are not comparable to the effort of the PAM data set used (e.g., seasonal trends for false killer whale, rough‐toothed dolphin, and *Kogia* spp.). The work presented here highlights the utility of using multiple methodologies for studying species in this region and others.

In terms of multiyear trends, results of this study at Hawai'i are novel for most species, though it is worth noting that, as the detection range covered by the HARPs is relatively small (2–5 km for most odontocetes), shifts in acoustic presence may represent small shifts in high‐use areas by island‐associated populations. In Hawai'i, few long‐term trends have been described, though portions of this data set have been used for long‐term assessments of sperm whales (Merkens et al., 2019) and humpback whales (Allen et al., 2021). Additionally, there are multiple species (e.g., rough‐toothed dolphin, *Kogia* spp.) where the records provided here have little to no comparison in previous literature. In that way, this study provides a useful starting point for other species‐specific studies of these animals. Future work employing these results in models considering environmental and anthropogenic parameters will help provide explanations for the patterns observed. These will be useful in the further understanding of these species habitats and behaviors and may help to explain any existing behavioral discrepancies between this and previous studies. These behaviors and movements are important to understand in the context of the Hawaiian ecosystem to which these odontocetes belong. The work completed emphasizes the merits of establishing baselines and comparing patterns of detections on fine temporal and spatial scales in marine top predator monitoring efforts. The patterns established in this study provide useful records to which additional studies of included species can be compared, allowing for documentation of regional differences in temporal behaviors which may be relevant for conservation purposes. These processes are necessary for management and conservation efforts of these species regionally as well as worldwide.

## AUTHOR CONTRIBUTIONS

**Morgan A. Ziegenhorn:** Conceptualization (lead); data processing and analysis (lead); metholodolgy (lead); validation (lead); visualization (lead); writing – original draft (lead); writing – review and editing (lead). **John A. Hildebrand:** Conceptualization (supporting); methodology (supporting); resources (equal); supervision (equal); writing – original draft (supporting); writing – review and editing (supporting). **Erin M. Oleson:** Data collection (lead); methodology (supporting); funding acquisition (equal); resources (equal); validation (equal); writing – review and editing (supporting). **Robin W. Baird:** Methodology (supporting); validation (equal); resources (supporting); writing – review and editing (supporting); **Sean M. Wiggins:** Resources (equal); writing – review and editing (supporting). **Simone Baumann‐Pickering:** Conceptualization (supporting); methodology (supporting); resources (equal); funding acquisition (equal); writing – original draft (supporting); writing – review and editing (supporting).

## CONFLICT OF INTEREST

The authors have no conflicts of interest to report.

## Supporting information


Appendix S1.

## Data Availability

Data are available on Dryad at: https://doi.org/10.5061/dryad.v15dv4209
