# Supplementary material for: Odontocete spatial patterns and temporal drivers of detection at sites in the Hawaiian islands
Source: Ecol Evol. 2023 Jan 6;13(1):e9688. doi: 10.1002/ece3.9688 (PMC9817196; doi:10.1002/ece3.9688)
Supplement: Supplementary file 1 — Appendix S1. [file ECE3-13-e9688-s001.docx]

**Appendix S1** *Recording schedule for deployments from all sites*.

| Deployment | Sampling Frequency (kHz) | Duty Cycle  (minutes on/ total minutes) | Latitude, Longitude  (decimal degrees) | Instrument Depth  (nearest 10 meters) | Start Date | End Date |
| --- | --- | --- | --- | --- | --- | --- |
| Hawaiʻi05 | 200 | 5/5 | 19.578, -156.014 | 550 | 2009-02-10 | 2009-04-01 |
| Hawaiʻi06 | 200 | 5/15 | 19.583, -156.016 | 750 | 2009-04-23 | 2009-08-18 |
| Hawaiʻi07 | 200 | none | 19.582, -156.015 | 720 | 2009-10-25 | 2009-12-15 |
| Hawaiʻi08 | 200 | 5/12 | 19.581, -156.015 | 750 | 2009-12-20 | 2010-03-05 |
| Hawaiʻi09 | 200 | none | 19.581, -156.016 | 750 | 2010-05-01 | 2010-06-16 |
| Hawaiʻi10 | 200 | 5/8 | 19.582, -156.015 | 720 | 2010-09-30 | 2011-03-12 |
| Hawaiʻi11 | 200 | 5/8 | 19.582, -156.015 | 720 | 2011-05-12 | 2011-10-22 |
| **Hawaiʻi16** | 200 | 5/15 | 19.583, -156.015 | 720 | 2013-10-23 | 2014-04-03 |
| **Hawaiʻi17** | 200 | none | 19.583, -156.016 | 720 | 2014-03-25 | 2014-07-14 |
| **Hawaiʻi18** | 320 | none | 19.583, -156.016 | 720 | 2014-07-28 | 2014-10-12 |
| **Hawaiʻi19** | 320 | none | 19.583, -156.016 | 720 | 2014-12-06 | 2015-03-06 |
| **Hawaiʻi20** | 320 | none | 19.583, -156.016 | 720 | 2015-04-25 | 2015-08-18 |
| **Hawaiʻi22** | 200 | none | 19.583, -156.016 | 720 | 2015-11-07 | 2016-03-19 |
| **Hawaiʻi23** | 200 | none | 19.583, -156.016 | 720 | 2016-07-04 | 2016-09-14 |
| **Hawaiʻi26** | 200 | none | 19.582, -156.015 | 740 | 2017-07-12 | 2017-10-25 |
| **Hawaiʻi27** | 200 | none | 19.583, -156.016 | 750 | 2017-10-26 | 2018-04-25 |
| **Hawaiʻi28** | 200 | none | 19.583, -156.016 | 730 | 2018-04-29 | 2018-11-19 |
| **Hawaiʻi29** | 200 | none | 19.583, -156.016 | 740 | 2018-11-23 | 2019-03-31 |
| Hawaiʻi30 | 200 | none | 19.583, -156.015 | 700 | 2019-04-04 | 2019-09-29 |
| Kaua**ʻ**i01 | 200 | 5/20 | 21.953, -159.887 | 720 | 2009-10-08 | 2010-05-13 |
| Kaua**ʻ**i02 | 200 | none | 21.954, -159.890 | 730 | 2010-06-04 | 2010-08-20 |
| **Kauaʻi05** | 200 | 5/7 | 21.949, -159.888 | 730 | 2016-07-09 | 2017-08-09 |
| Manawai01 | 200 | 5/20 | 27.725, -175.638 | 770 | 2009-10-20 | 2010-05-24 |
| Manawai02 | 200 | none | 27.727, -175.632 | 750 | 2010-06-01 | 2010-09-17 |
| Manawai04 | 200 | none | 27.725, -175.638 | 770 | 2011-04-12 | 2011-07-29 |
| Manawai05 | 200 | 5/8 | 27.725, -175.638 | 770 | 2011-08-15 | 2012-01-07 |
| **Manawai08** | 200 | 5/20 | 27.742, -175.559 | 970 | 2014-09-12 | 2015-07-16 |
| Manawai09 | 200 | 5/30 | 27.742, -175.560 | 960 | 2015-10-15 | 2016-08-14 |
| Manawai10 | 200 | 5/30 | 27.741, -175.560 | 960 | 2016-08-20 | 2017-03-14 |

**Table S1.1. Recording schedule.** Recording schedule for deployments from all sites, adapted from Ziegenhorn et al. (2022). Deployments with a 25 kHz crossover between the low and high frequency hydrophones have been bolded. Lines in-between rows represent different sites, including Manawai subsites 1-2.

**Appendix S2** *Seasonal species composition for each site*


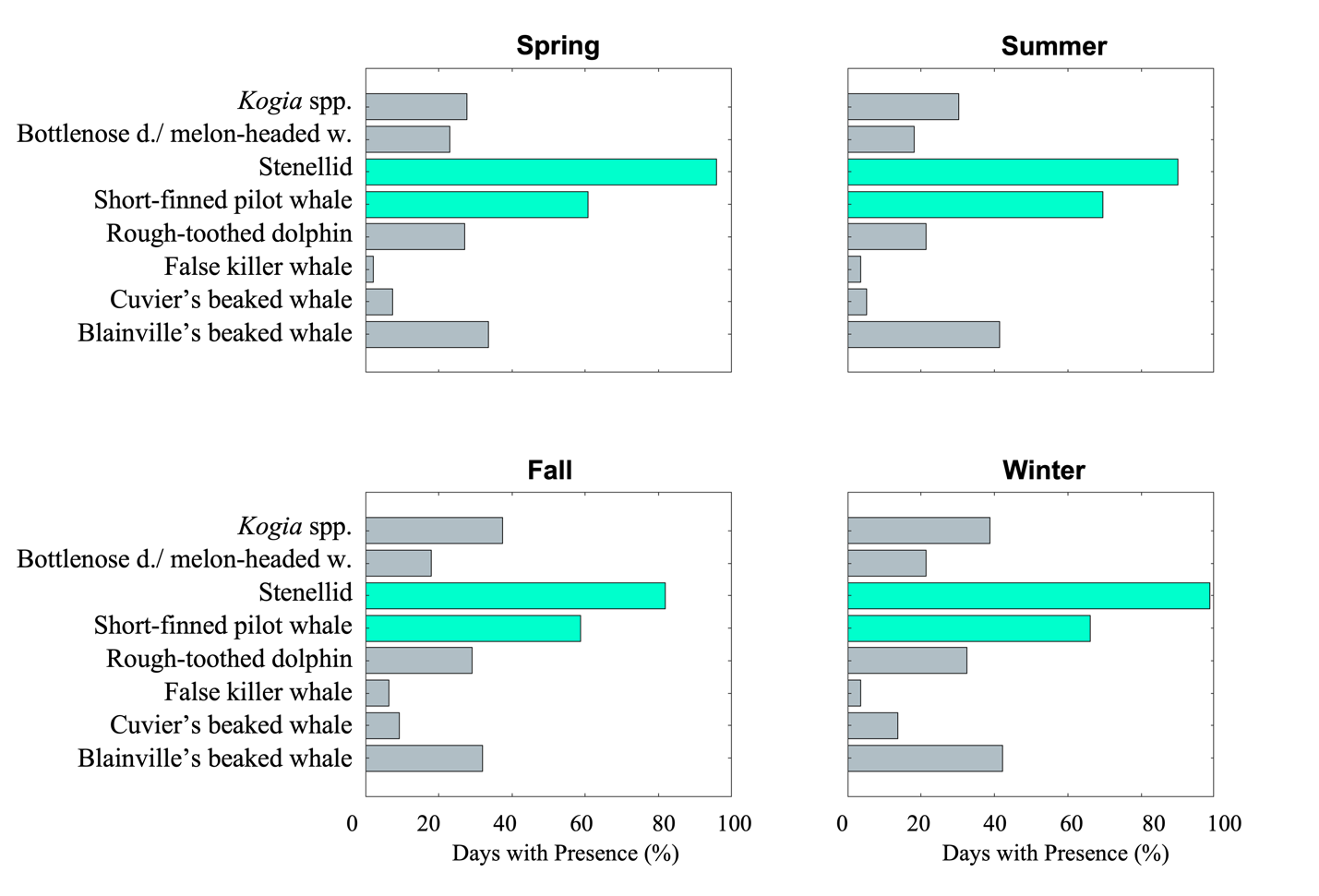


**Figure S2.1. Percentage days with presence-Hawaiʻi.** Percent days out of total recording days at Hawaiʻi with presence of each type for each season. Values greater than 50% are shown in green.

**
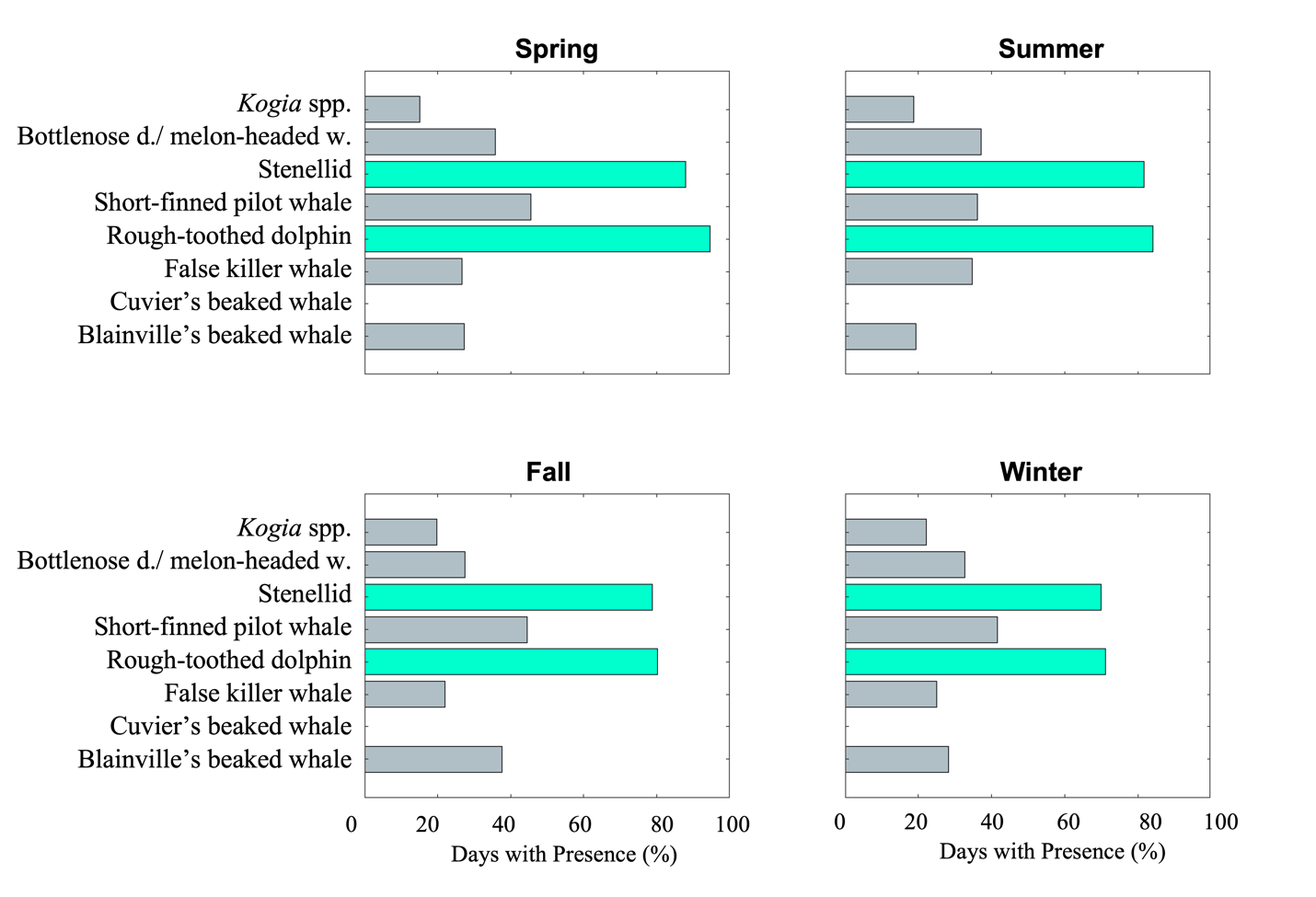
**

**Figure S2.2. Percentage days with presence-Kauaʻi.** Percent days out of total recording days at Kauaʻi with presence of each type for each season. Values greater than 50% are shown in green.

**
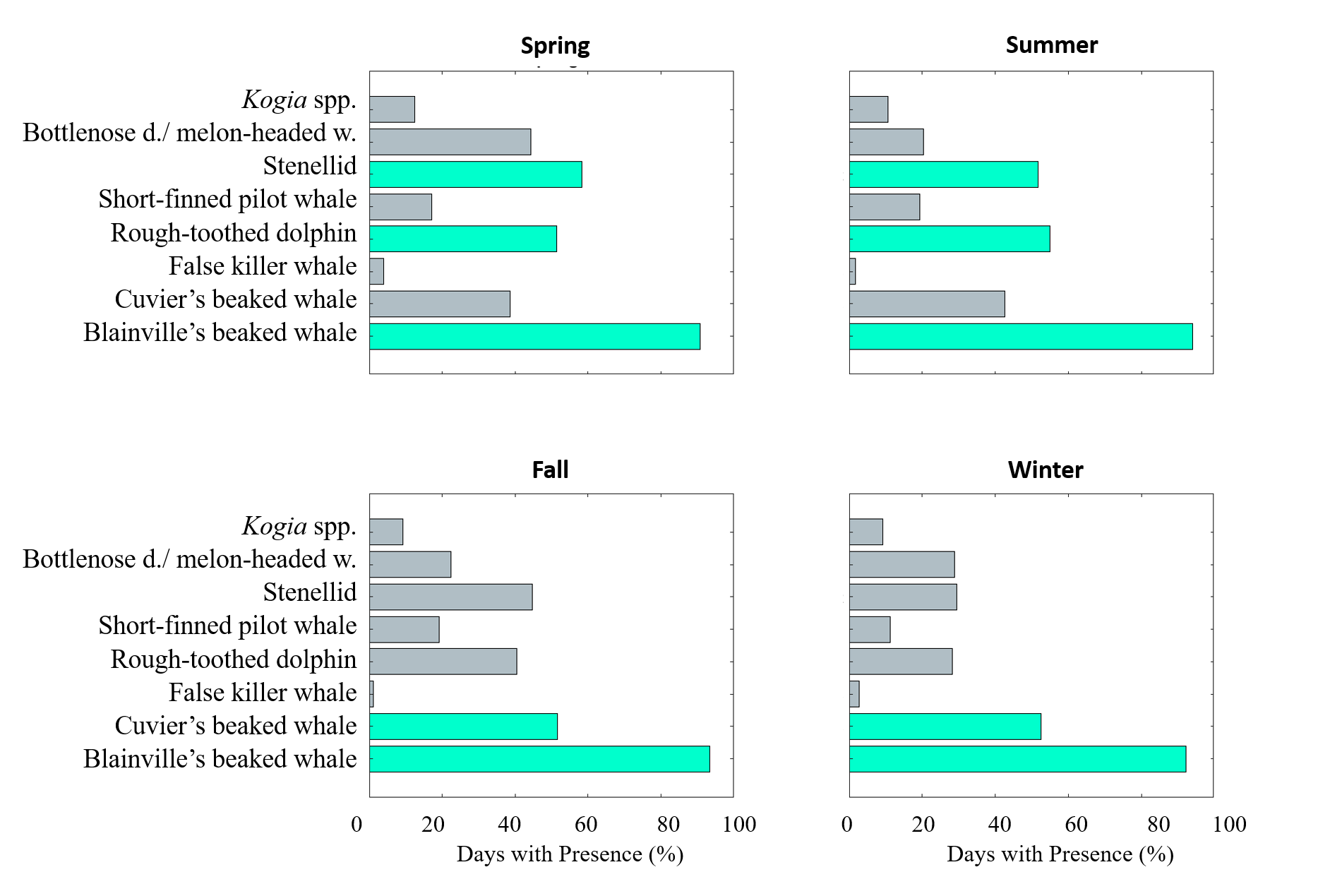
**

**Figure S2.3. Percentage days with presence-Manawai.** Percent days out of total recording days at Manawai with presence of each type for each season. Values greater than 50% are shown in green.

**Appendix S3** *Full timeseries of species presence*

**
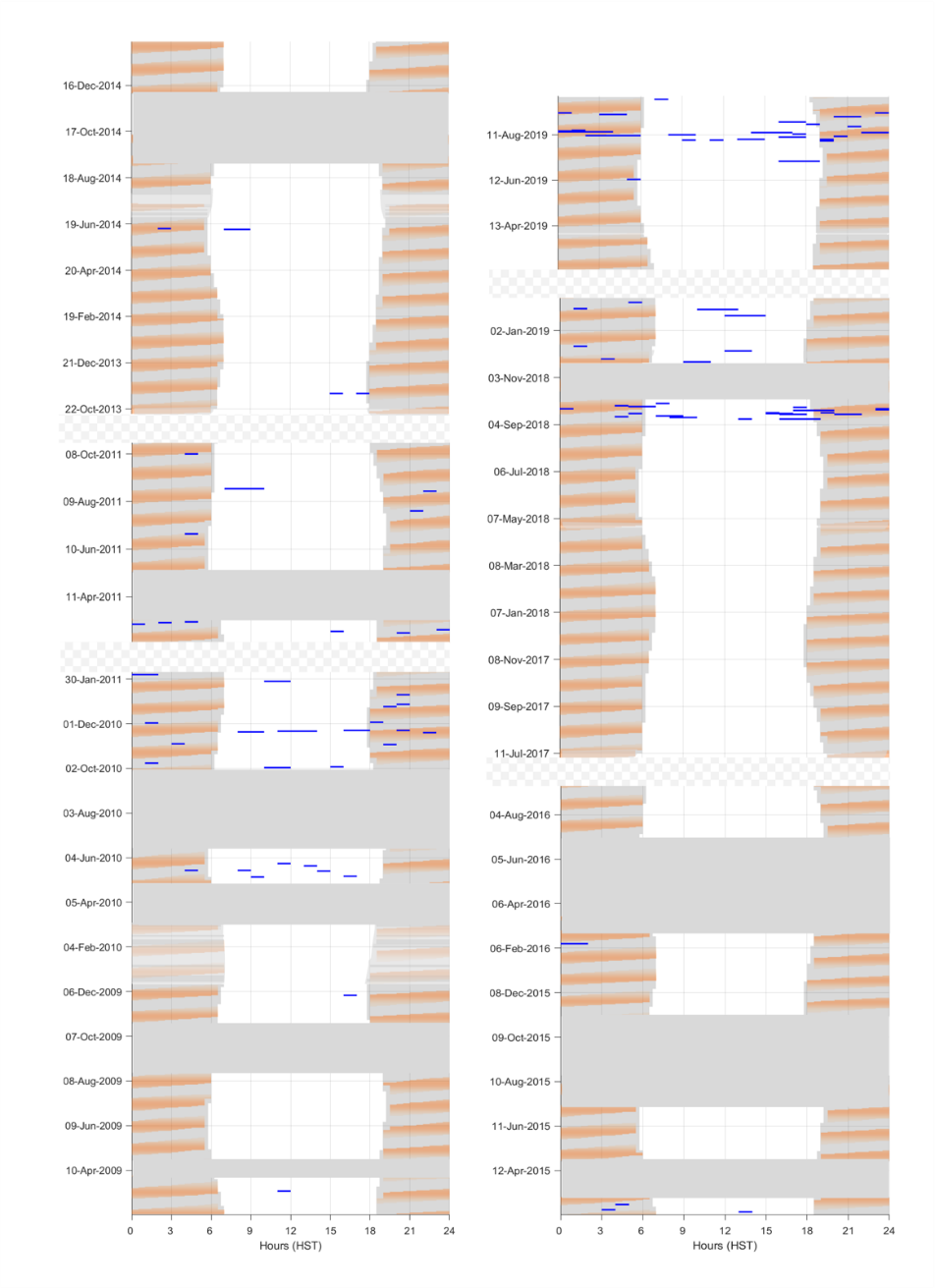
**

**Figure S3.1. Diel and lunar timeseries for false killer whales at Hawaiʻi.** Hours of presence for false killer whales at Hawaiʻi (blue). Date is shown on the y-axis, and hour of day on the x-axis. Lunar illumination (orange shading), nighttime hours (grey shading), times of no effort (dark gray boxes), and large time gaps (hashed gray boxes) are drawn.

**
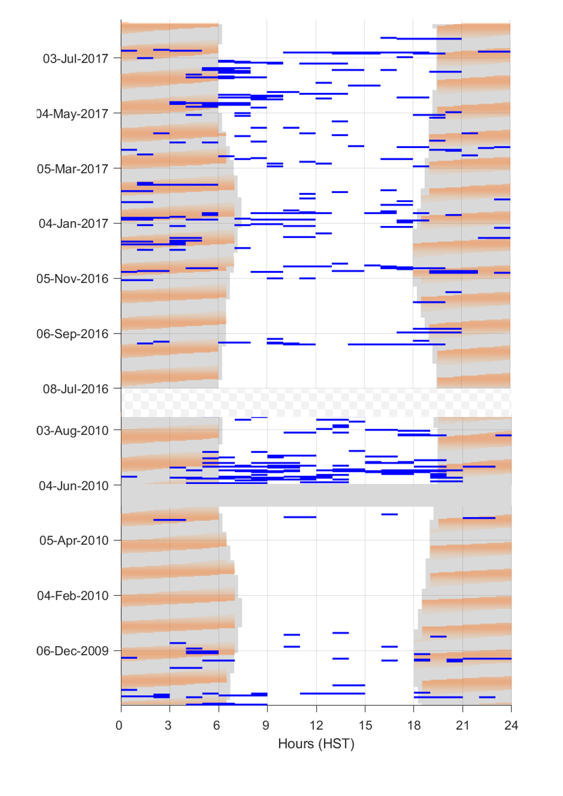
**

**Figure S3.2. Diel/lunar timeseries- false killer whales at Kauaʻi.** Hours of presence for false killer whales at Kauaʻi (blue). Date is shown on the y-axis, and hour of day on the x-axis. Lunar illumination (orange shading), nighttime hours (grey shading), times of no effort (dark gray boxes), and large time gaps (hashed gray boxes) are drawn.


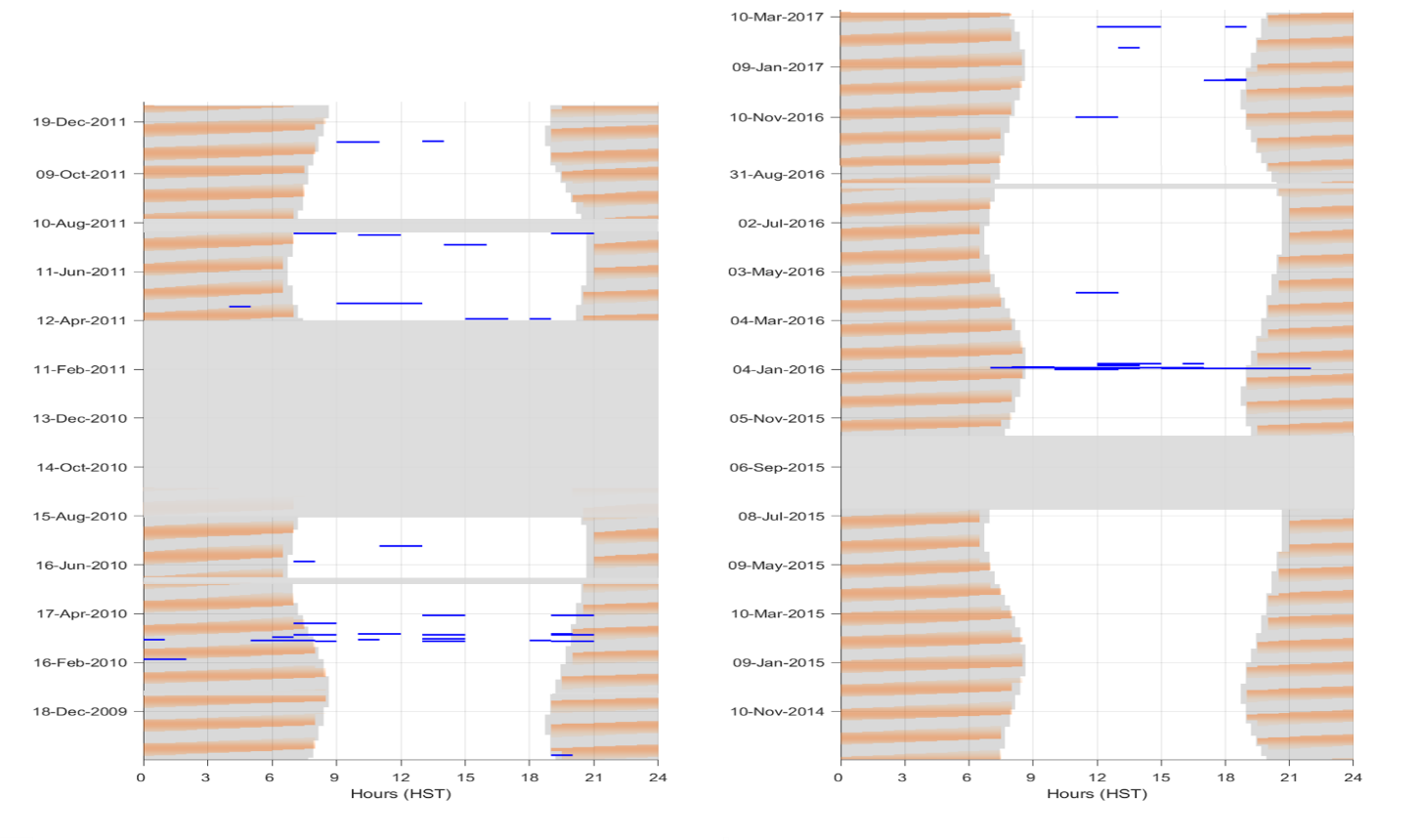


**Figure S3.3. Diel/lunar timeseries- false killer whales at Manawai.** Hours of presence for false killer whales (blue) at Manawai. Date is shown on the y-axis, and hour of day on the x-axis. Lunar illumination (orange shading), nighttime hours (grey shading), times of no effort (dark gray boxes), and large time gaps (hashed gray boxes) are drawn.

**
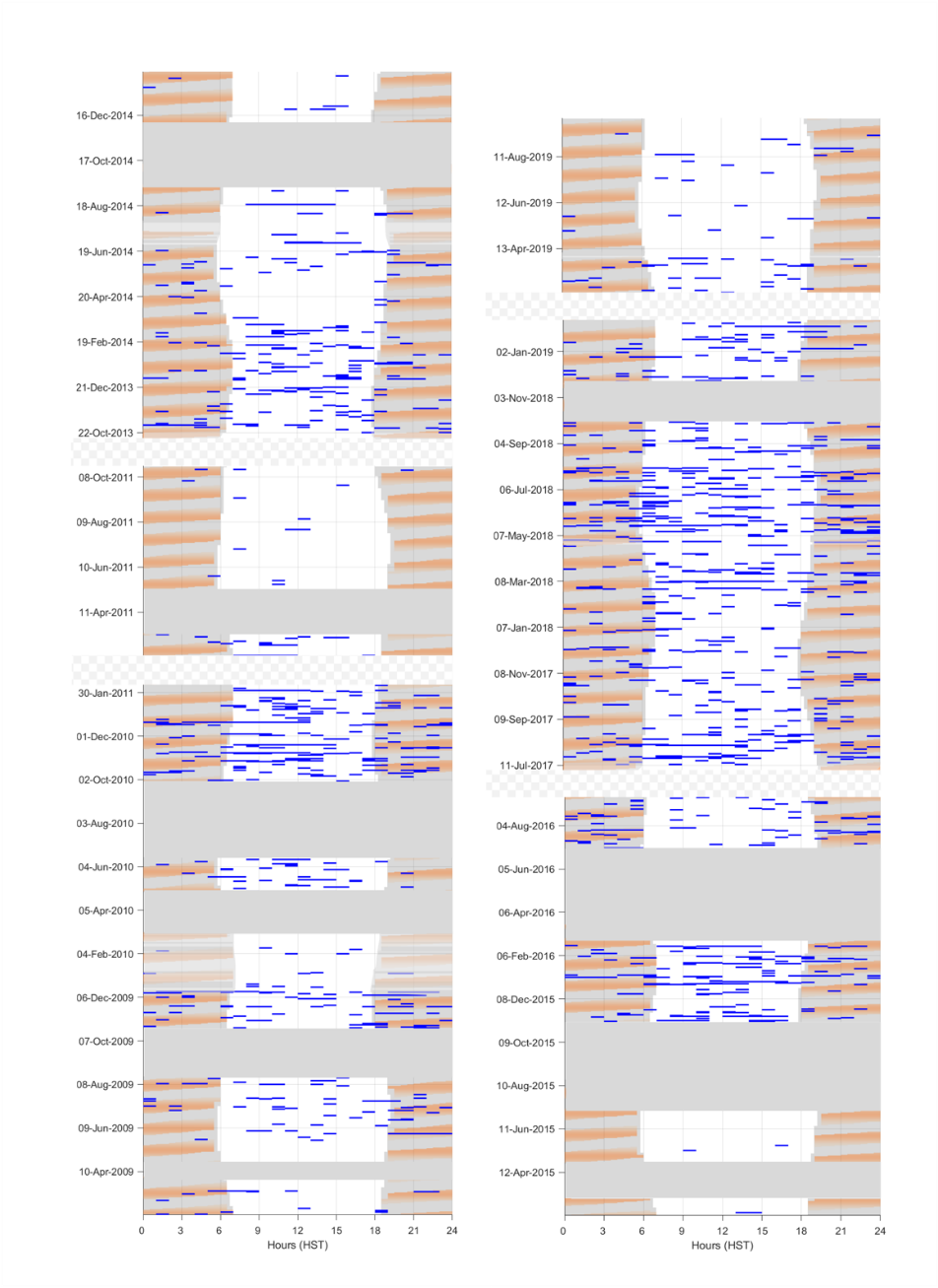
**

**Figure S3.4. Diel/lunar timeseries- *Kogia* spp. at Hawaiʻi.** Hours of presence for *Kogia* spp. at Hawaiʻi (blue). Date is shown on the y-axis, and hour of day on the x-axis. Lunar illumination (orange shading), nighttime hours (grey shading), times of no effort (dark gray boxes), and large time gaps (hashed gray boxes) are drawn.

**
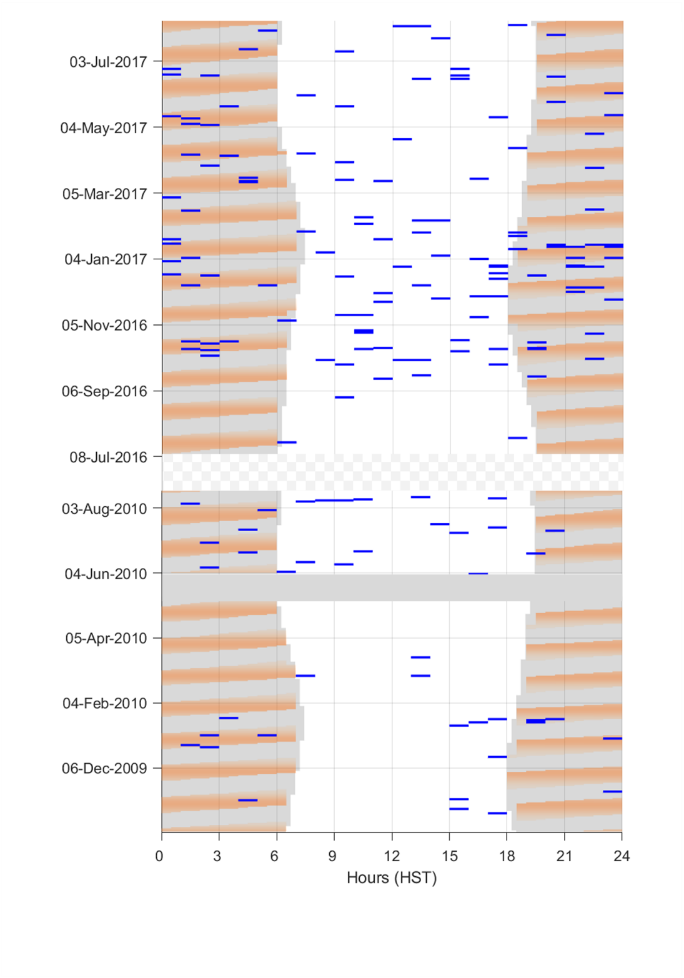
**

**Figure S3.5. Diel/lunar timeseries- *Kogia* spp. at Kauaʻi.** Hours of presence for *Kogia* spp. at Kauaʻi (blue). Date is shown on the y-axis, and hour of day on the x-axis. Lunar illumination (orange shading), nighttime hours (grey shading), times of no effort (dark gray boxes), and large time gaps (hashed gray boxes) are drawn.


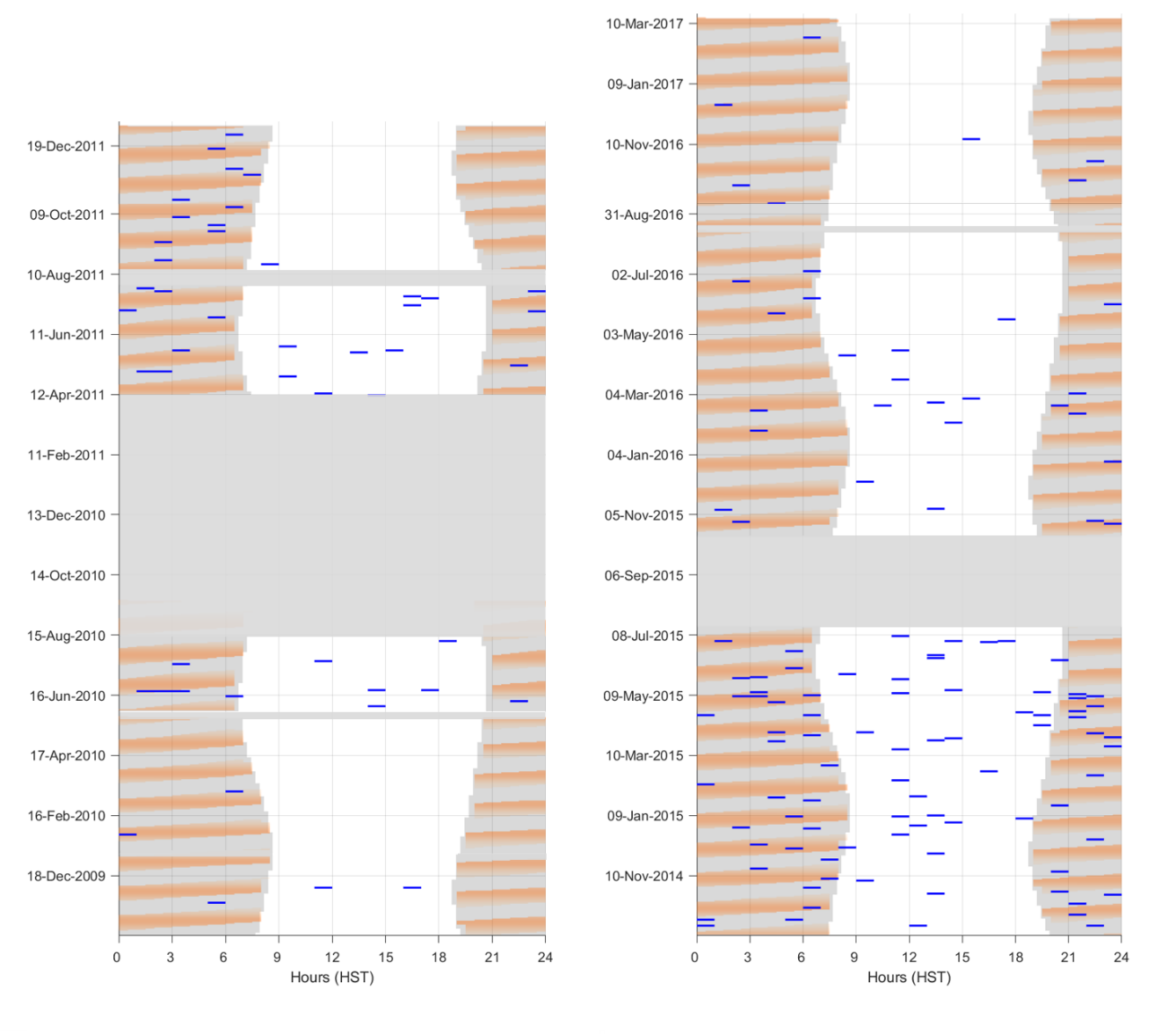


**Figure S3.6. Diel/lunar timeseries- *Kogia* spp. at Manawai.** Hours of presence for *Kogia* spp. (blue) at Manawai. Date is shown on the y-axis, and hour of day on the x-axis. Lunar illumination (orange shading), nighttime hours (grey shading), times of no effort (dark gray boxes), and large time gaps (hashed gray boxes) are drawn.


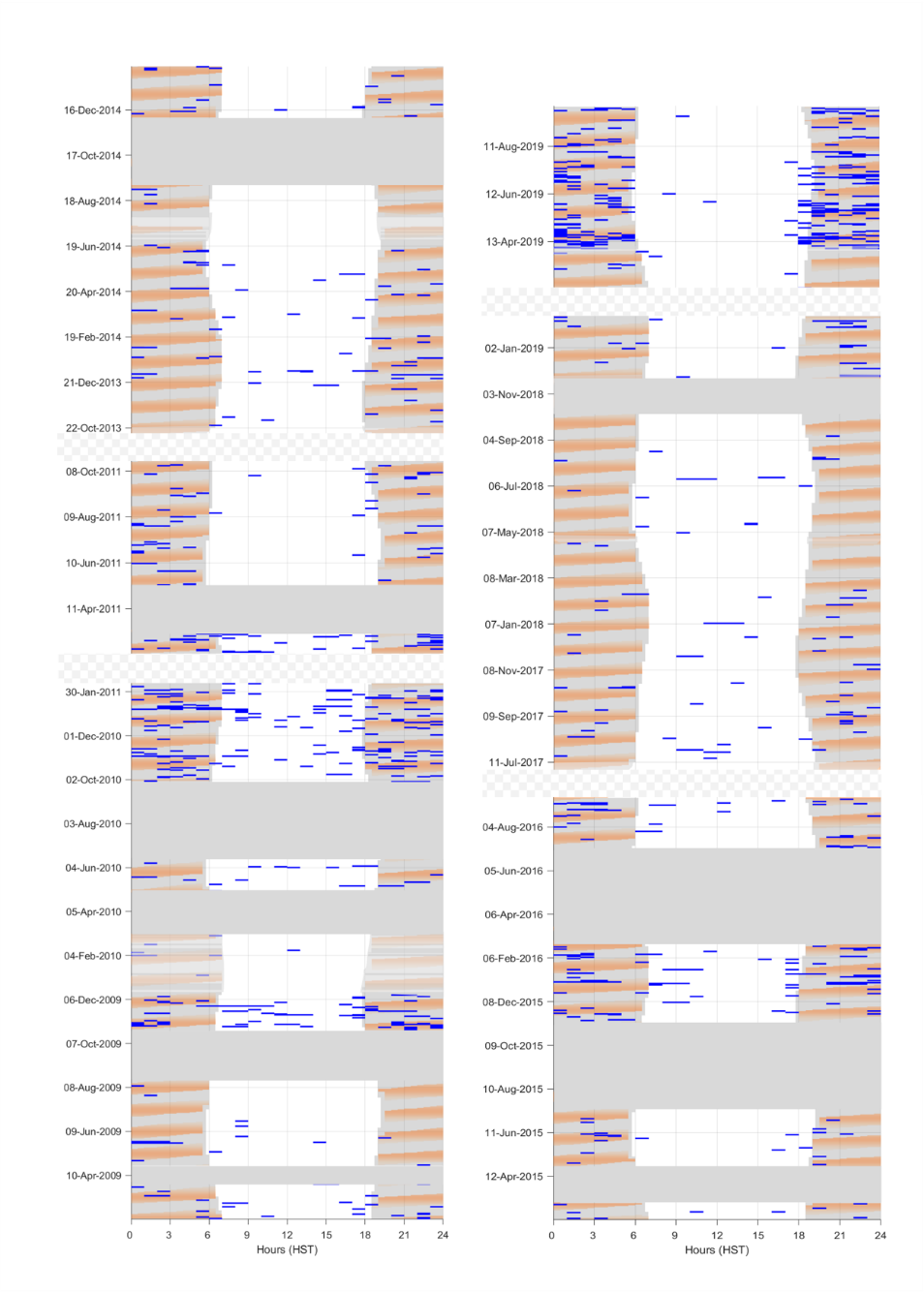


**Figure S3.7. Diel/lunar timeseries- rough-toothed dolphin at Hawaiʻi.** Hours of presence for rough-toothed dolphin at Hawaiʻi (blue). Date is shown on the y-axis, and hour of day on the x-axis. Lunar illumination (orange shading), nighttime hours (grey shading), times of no effort (dark gray boxes), and large time gaps (hashed gray boxes) are drawn.


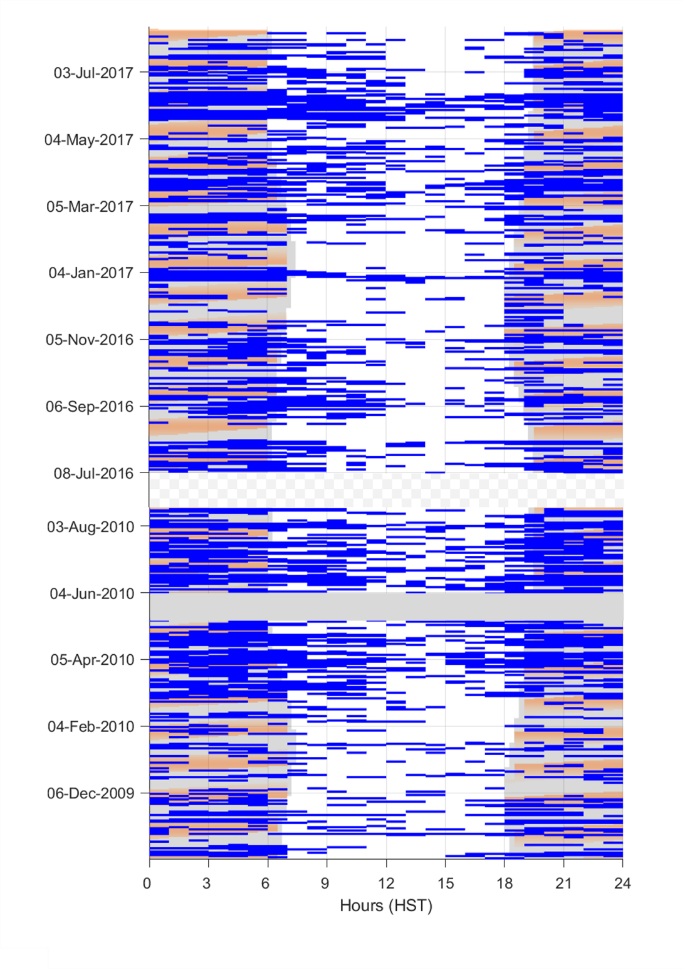


**Figure S3.8. Diel/lunar timeseries- rough-toothed dolphin at Kauaʻi.** Hours of presence for rough-toothed dolphin at Kauaʻi (blue). Date is shown on the y-axis, and hour of day on the x-axis. Lunar illumination (orange shading), nighttime hours (grey shading), times of no effort (dark gray boxes), and large time gaps (hashed gray boxes) are drawn.


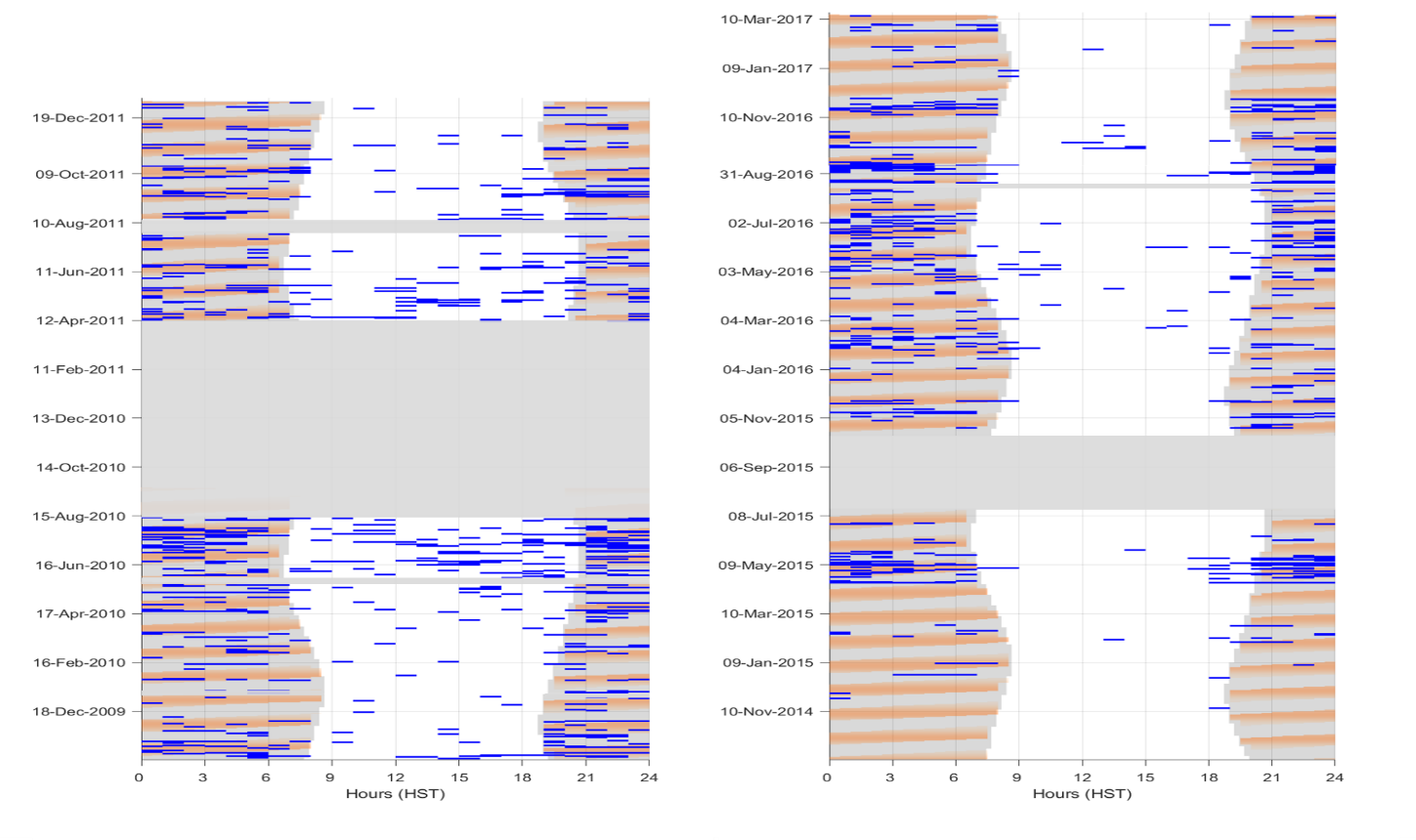


**Figure S3.9. Diel/lunar timeseries- rough-toothed dolphin at Manawai.** Hours of presence for rough-toothed dolphin (blue) at Manawai. Date is shown on the y-axis, and hour of day on the x-axis. Lunar illumination (orange shading), nighttime hours (grey shading), times of no effort (dark gray boxes), and large time gaps (hashed gray boxes) are drawn.


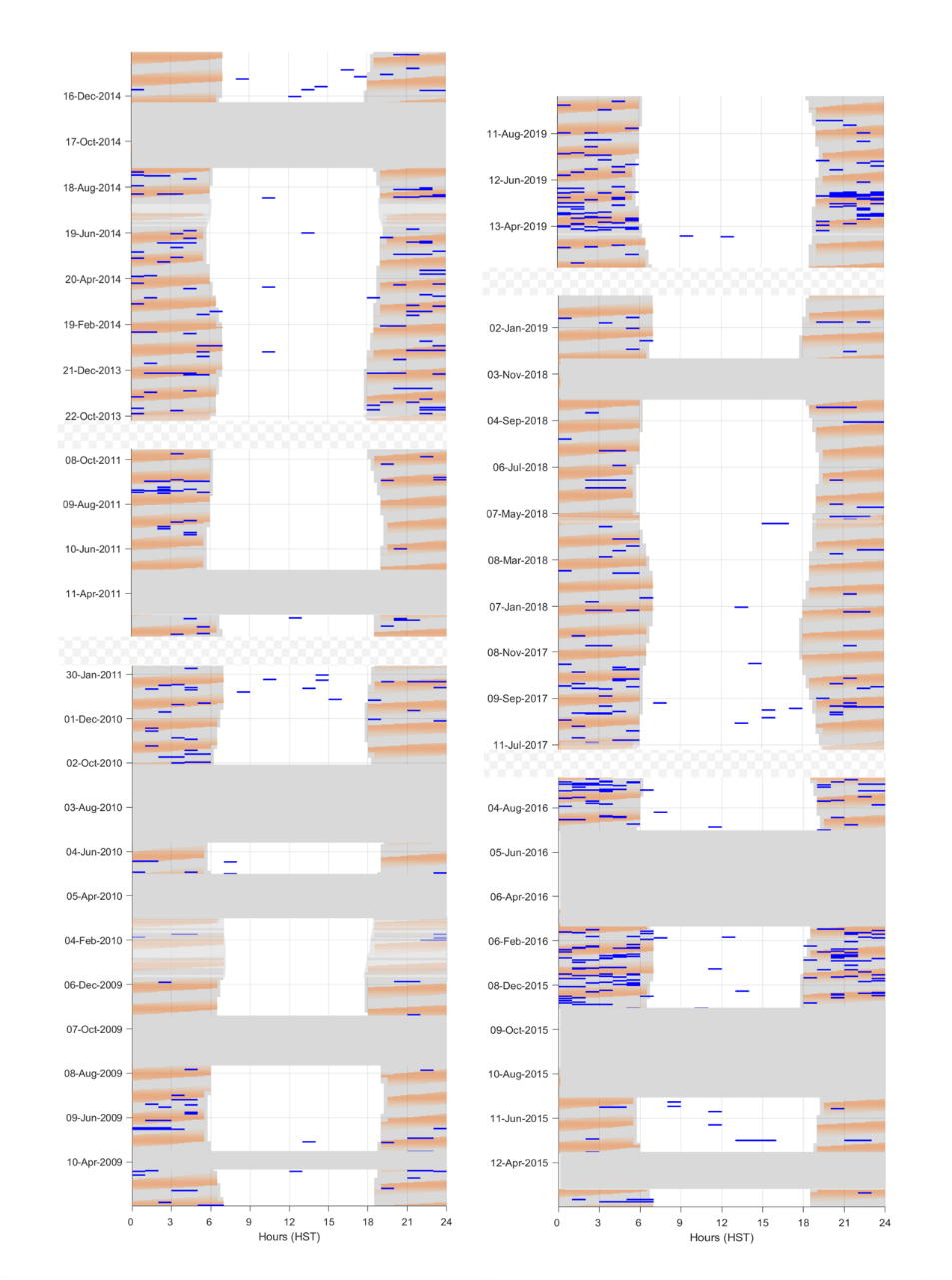


**Figure S3.10. Diel/lunar timeseries- bottlenose dolphin and melon-headed whale at Hawaiʻi.** Hours of presence for bottlenose dolphin/melon-headed whale at Hawaiʻi (blue). Date is shown on the y-axis, and hour of day on the x-axis. Lunar illumination (orange shading), nighttime hours (grey shading), times of no effort (dark gray boxes), and large time gaps (hashed gray boxes) are drawn.

**
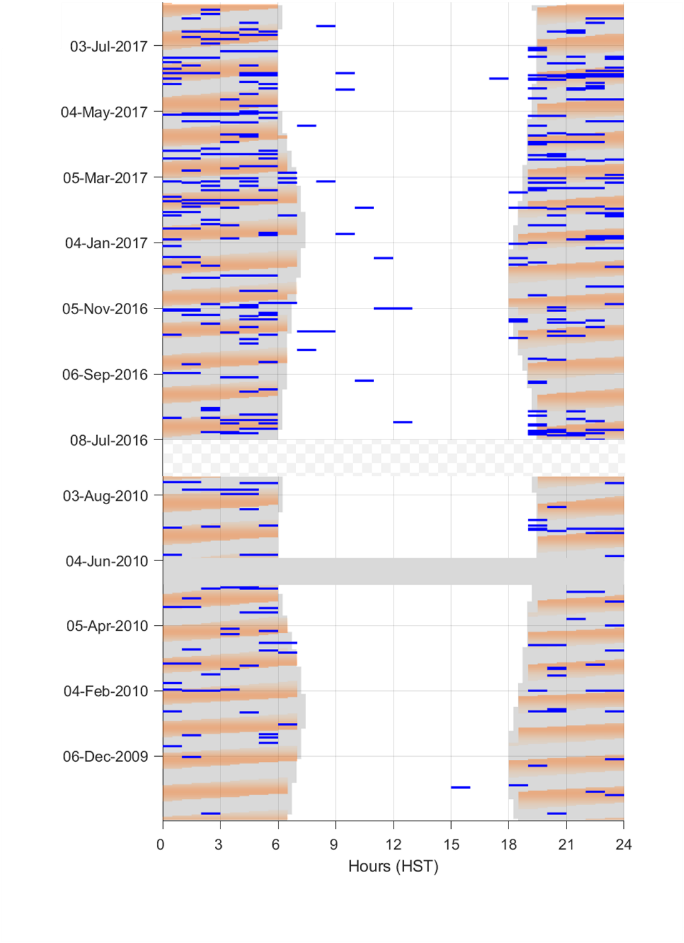
**

**Figure S3.11. Diel/lunar timeseries- bottlenose dolphin and melon-headed whale at Kauaʻi.** Hours of presence for bottlenose dolphin/melon-headed whale at Kauaʻi (blue). Date is shown on the y-axis, and hour of day on the x-axis. Lunar illumination (orange shading), nighttime hours (grey shading), times of no effort (dark gray boxes), and large time gaps (hashed gray boxes) are drawn.


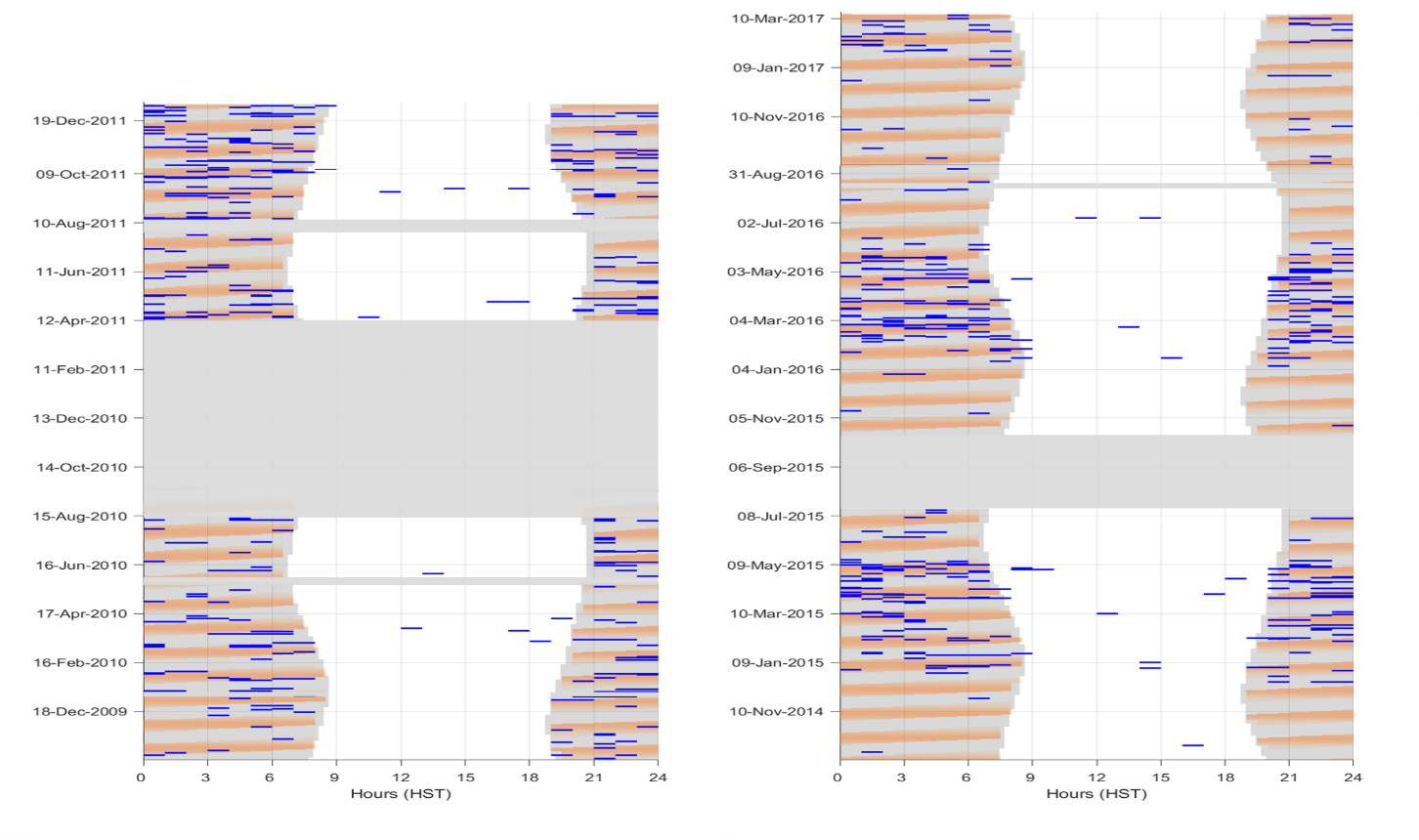


**Figure S3.12. Diel/lunar timeseries- bottlenose dolphin and melon-headed whale at Manawai.** Hours of presence for bottlenose dolphin/melon-headed whale (blue) at Manawai. Date is shown on the y-axis, and hour of day on the x-axis. Lunar illumination (orange shading), nighttime hours (grey shading), times of no effort (dark gray boxes), and large time gaps (hashed gray boxes) are drawn.


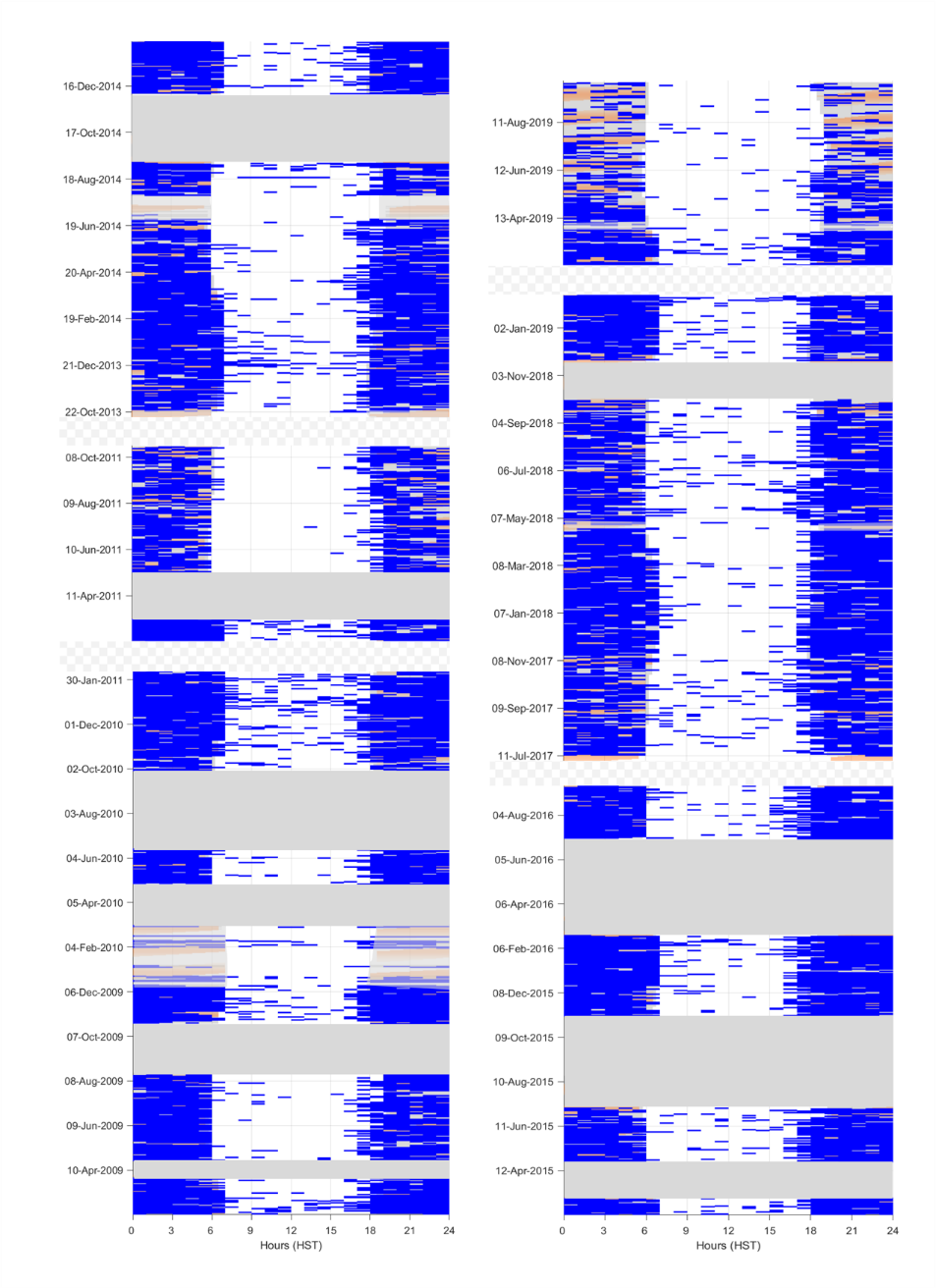


**Figure S3.13. Diel/lunar timeseries- stenellid dolphins at Hawaiʻi.** Hours of presence for stenellid dolphins at Hawaiʻi (blue). Date is shown on the y-axis, and hour of day on the x-axis. Lunar illumination (orange shading), nighttime hours (grey shading), times of no effort (dark gray boxes), and large time gaps (hashed gray boxes) are drawn.


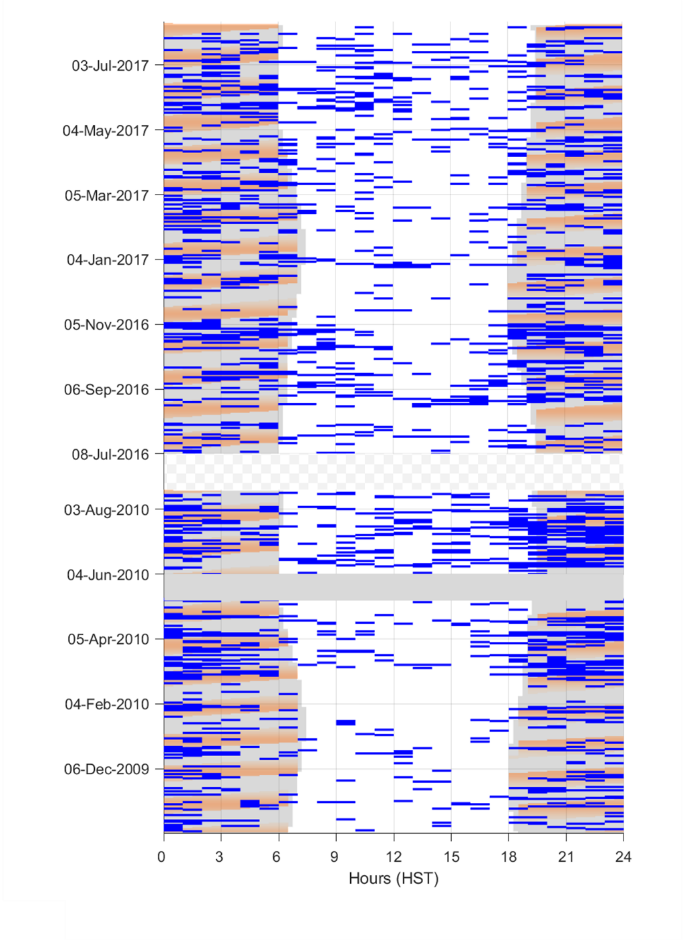


**Figure S3.14. Diel/lunar timeseries- stenellid dolphins at Kauaʻi.** Hours of presence for stenellid dolphins at Kauaʻi (blue). Date is shown on the y-axis, and hour of day on the x-axis. Lunar illumination (orange shading), nighttime hours (grey shading), times of no effort (dark gray boxes), and large time gaps (hashed gray boxes) are drawn.

**
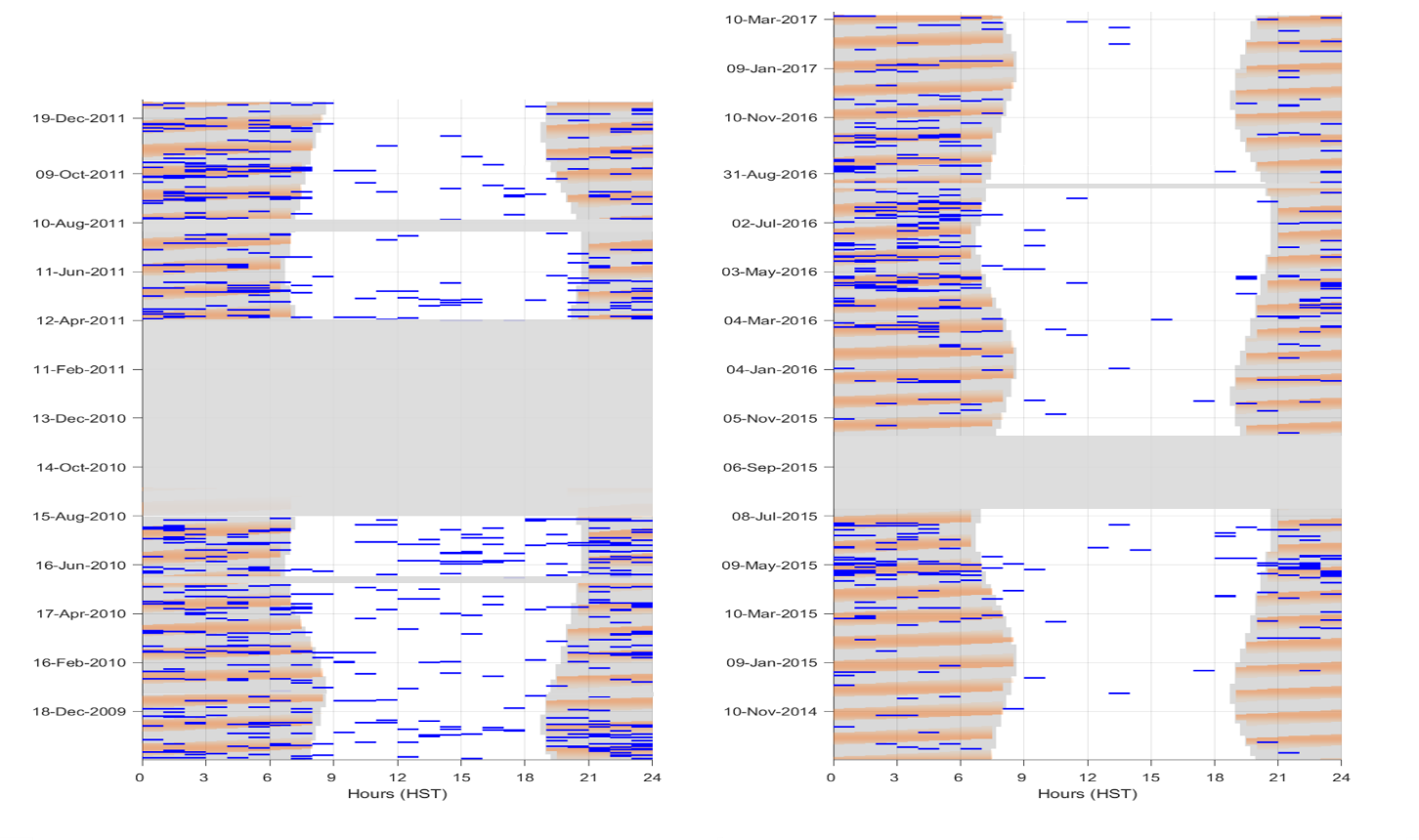
**

**Figure S3.15. Diel/lunar timeseries- stenellid dolphins at Manawai.** Hours of presence for stenellid dolphins (blue) at Manawai. Date is shown on the y-axis, and hour of day on the x-axis. Lunar illumination (orange shading), nighttime hours (grey shading), times of no effort (dark gray boxes), and large time gaps (hashed gray boxes) are drawn.


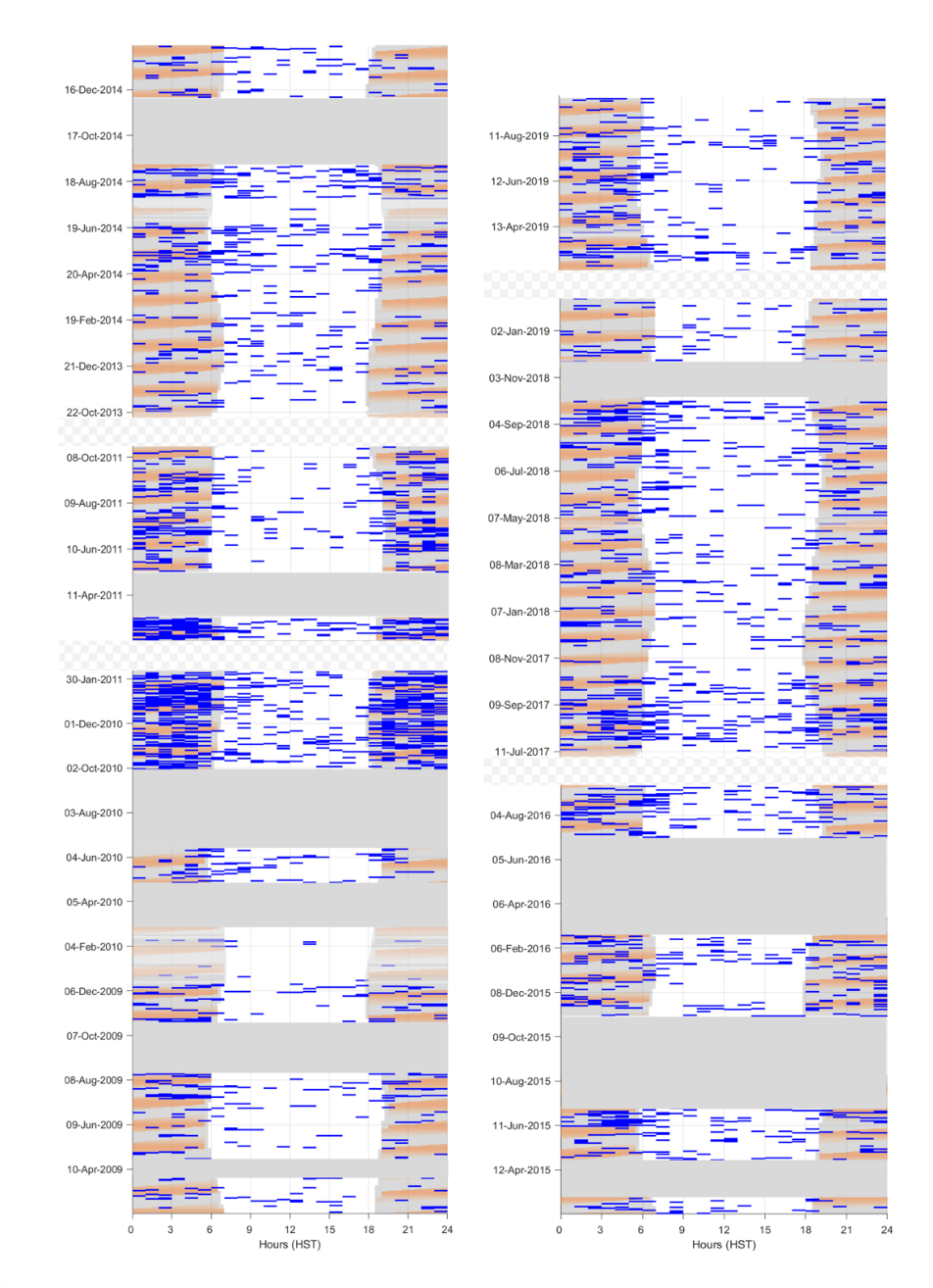


**Figure S3.16. Diel/lunar timeseries- short-finned pilot whales at Hawaiʻi.** Hours of presence for short-finned pilot whales at Hawaiʻi (blue). Date is shown on the y-axis, and hour of day on the x-axis. Lunar illumination (orange shading), nighttime hours (grey shading), times of no effort (dark gray boxes), and large time gaps (hashed gray boxes) are drawn.

**
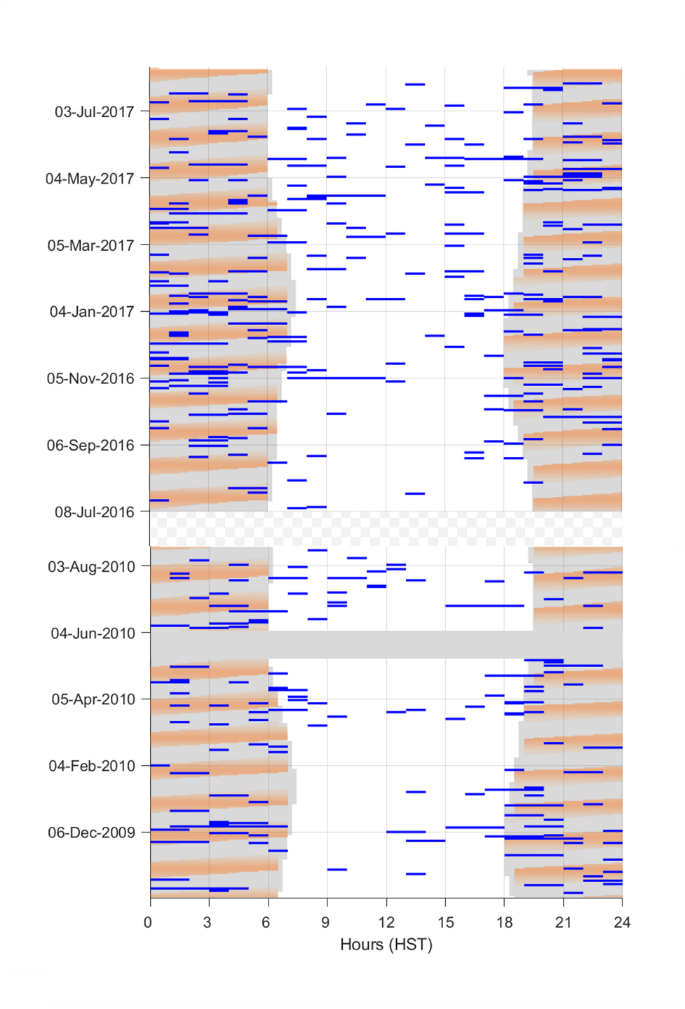
**

**F**

**Figure S3.17. Diel/lunar timeseries- short-finned pilot whales at Kauaʻi.** Hours of presence for short-finned pilot whales at Kauaʻi (blue). Date is shown on the y-axis, and hour of day on the x-axis. Lunar illumination (orange shading), nighttime hours (grey shading), times of no effort (dark gray boxes), and large time gaps (hashed gray boxes) are drawn.


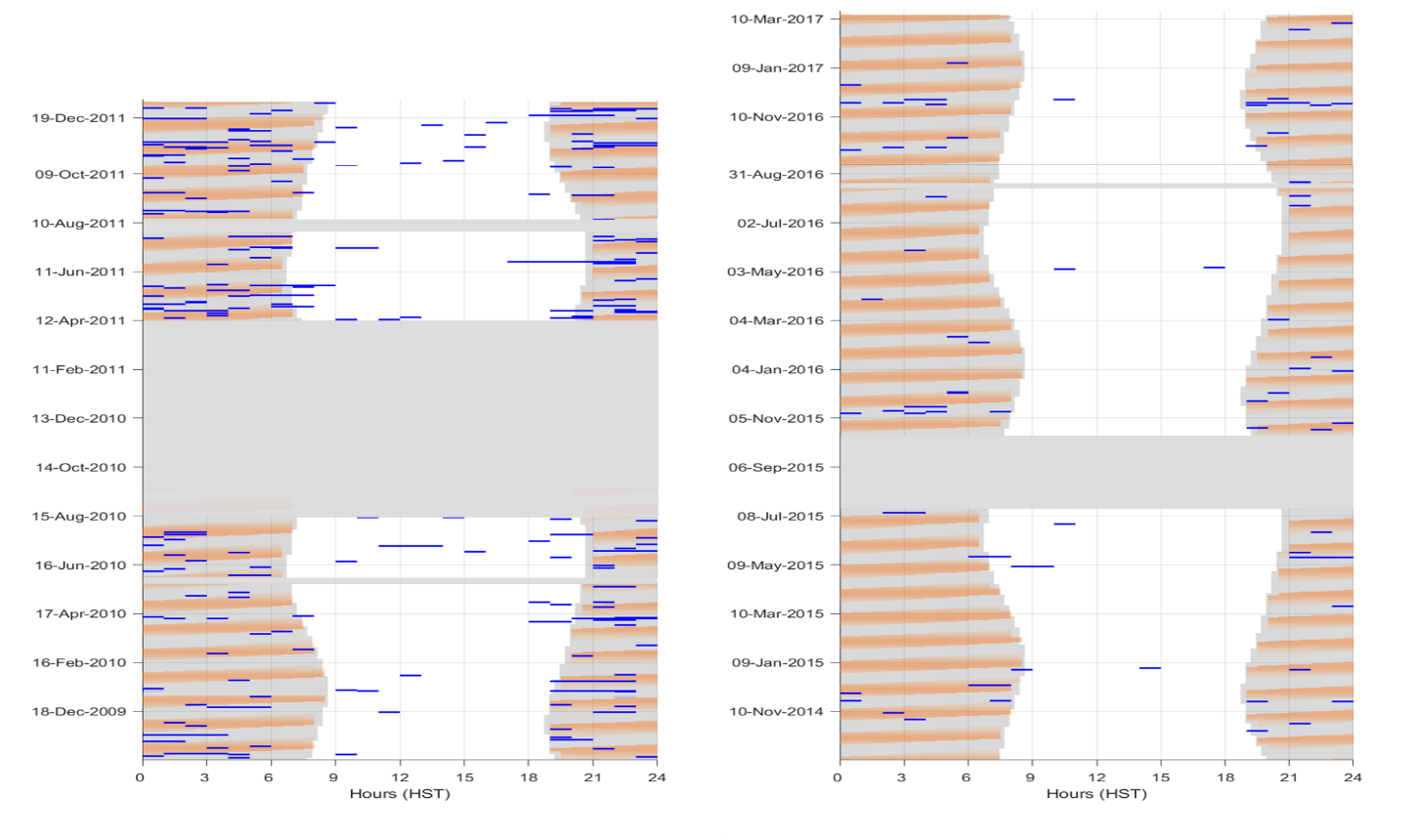


**Figure S3.18. Diel/lunar timeseries- short-finned pilot whales at Manawai.** Hours of presence for short-finned pilot whales (blue) at Manawai. Date is shown on the y-axis, and hour of day on the x-axis. Lunar illumination (orange shading), nighttime hours (grey shading), times of no effort (dark gray boxes), and large time gaps (hashed gray boxes) are drawn.

**
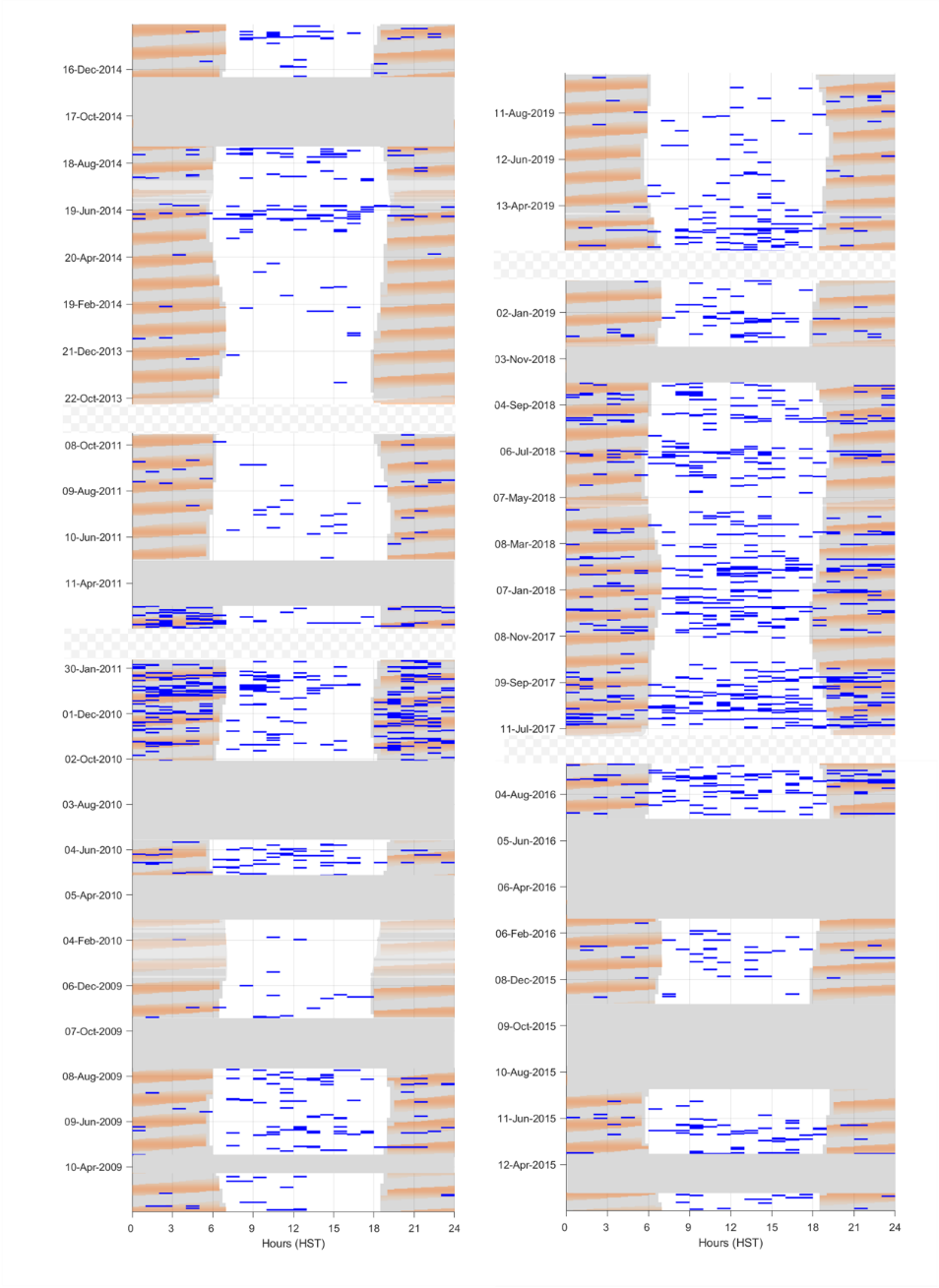
**

**Figure S3.19. Diel/lunar timeseries- Blainville’s beaked whales at Hawaiʻi.** Hours of presence for Blainville’s beaked whales at Hawaiʻi (blue). Date is shown on the y-axis, and hour of day on the x-axis. Lunar illumination (orange shading), nighttime hours (grey shading), times of no effort (dark gray boxes), and large time gaps (hashed gray boxes) are drawn.

**
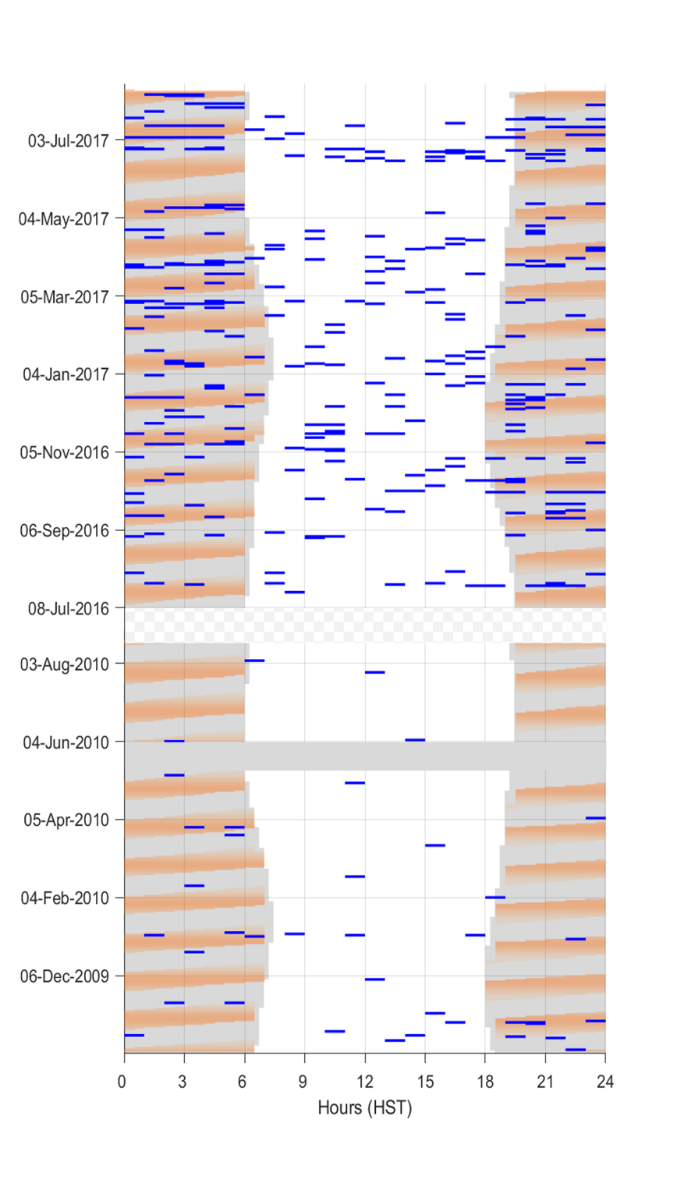
**

**Figure S3.20. Diel/lunar timeseries- Blainville’s beaked whales at Kauaʻi.** Hours of presence for Blainville’s beaked whales at Kauaʻi (blue). Date is shown on the y-axis, and hour of day on the x-axis. Lunar illumination (orange shading), nighttime hours (grey shading), times of no effort (dark gray boxes), and large time gaps (hashed gray boxes) are drawn.


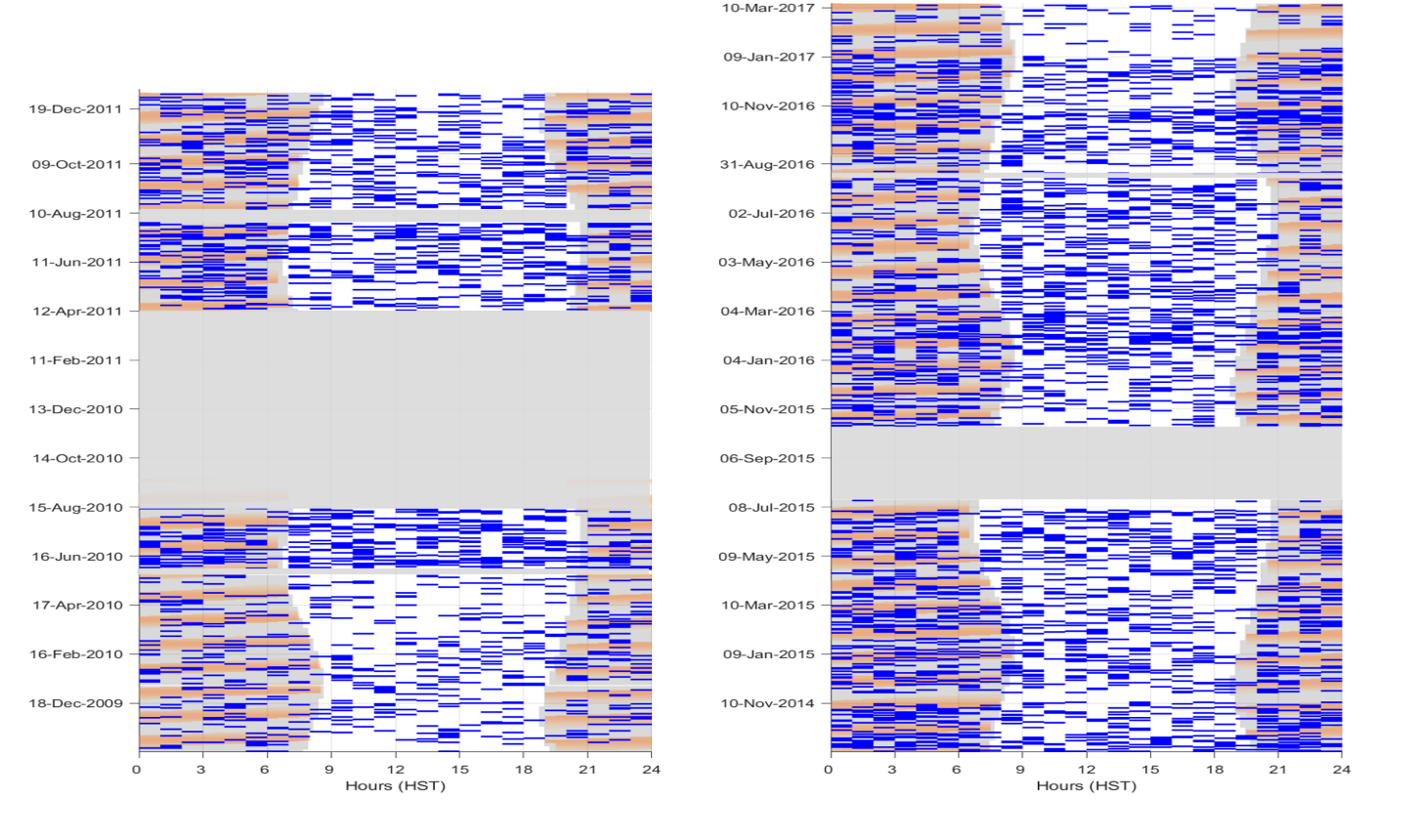


**Figure S3.21. Diel/lunar timeseries- Blainville’s beaked whales at Manawai.** Hours of presence for Blainville’s beaked whales (blue) at Manawai. Date is shown on the y-axis, and hour of day on the x-axis. Lunar illumination (orange shading), nighttime hours (grey shading), times of no effort (dark gray boxes), and large time gaps (hashed gray boxes) are drawn.

**
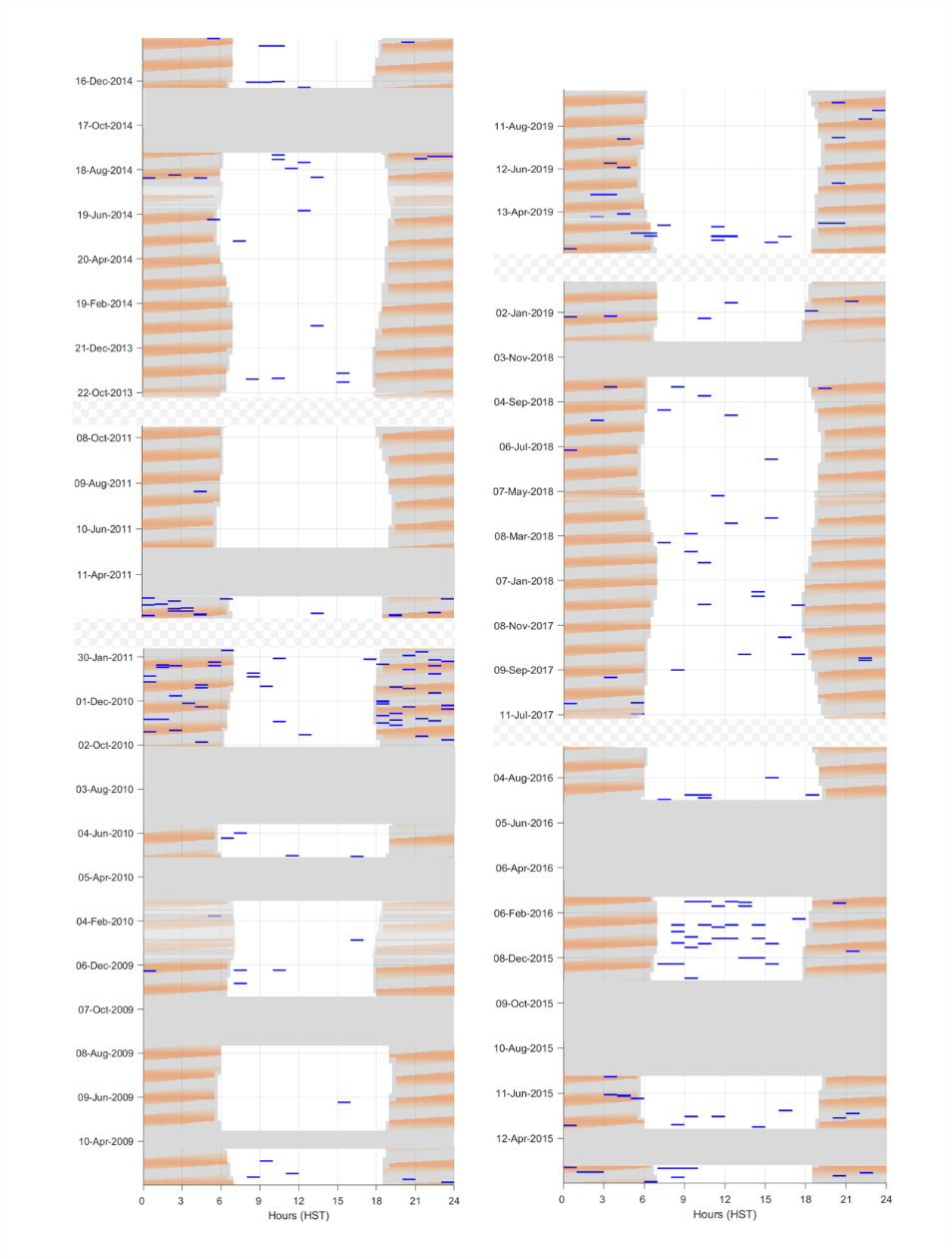
**

**Figure S3.22. Diel/lunar timeseries- Cuvier’s beaked whales at Hawaiʻi.** Hours of presence for Cuvier’s beaked whales at Hawaiʻi (blue). Date is shown on the y-axis, and hour of day on the x-axis. Lunar illumination (orange shading), nighttime hours (grey shading), times of no effort (dark gray boxes), and large time gaps (hashed gray boxes) are drawn.

**
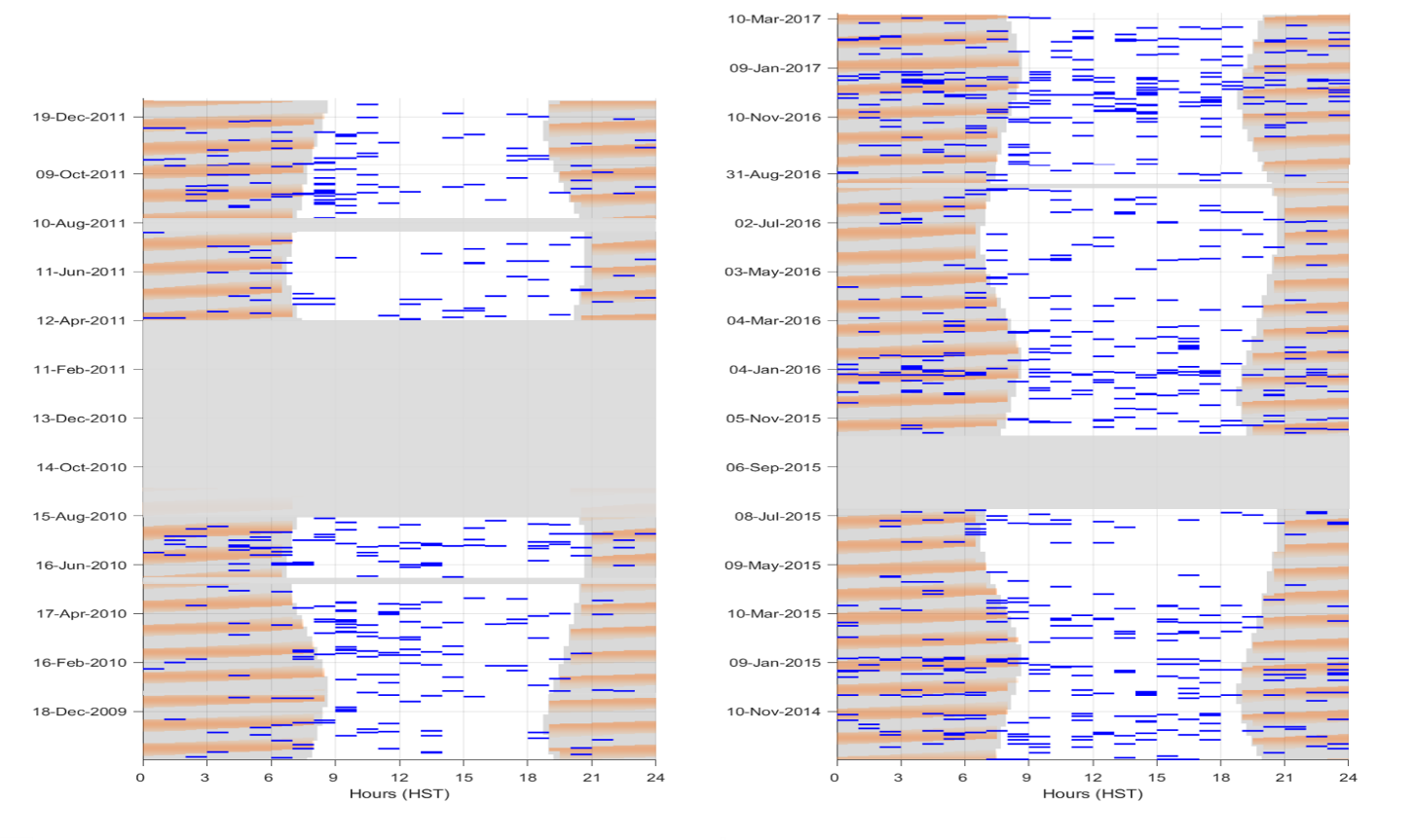
**

**Figure S3.23. Diel/lunar timeseries- Cuvier’s beaked whales at Manawai.** Hours of presence for Cuvier’s beaked whales (blue) at Manawai. Date is shown on the y-axis, and hour of day on the x-axis. Lunar illumination (orange shading), nighttime hours (grey shading), times of no effort (dark gray boxes), and large time gaps (hashed gray boxes) are drawn.

**
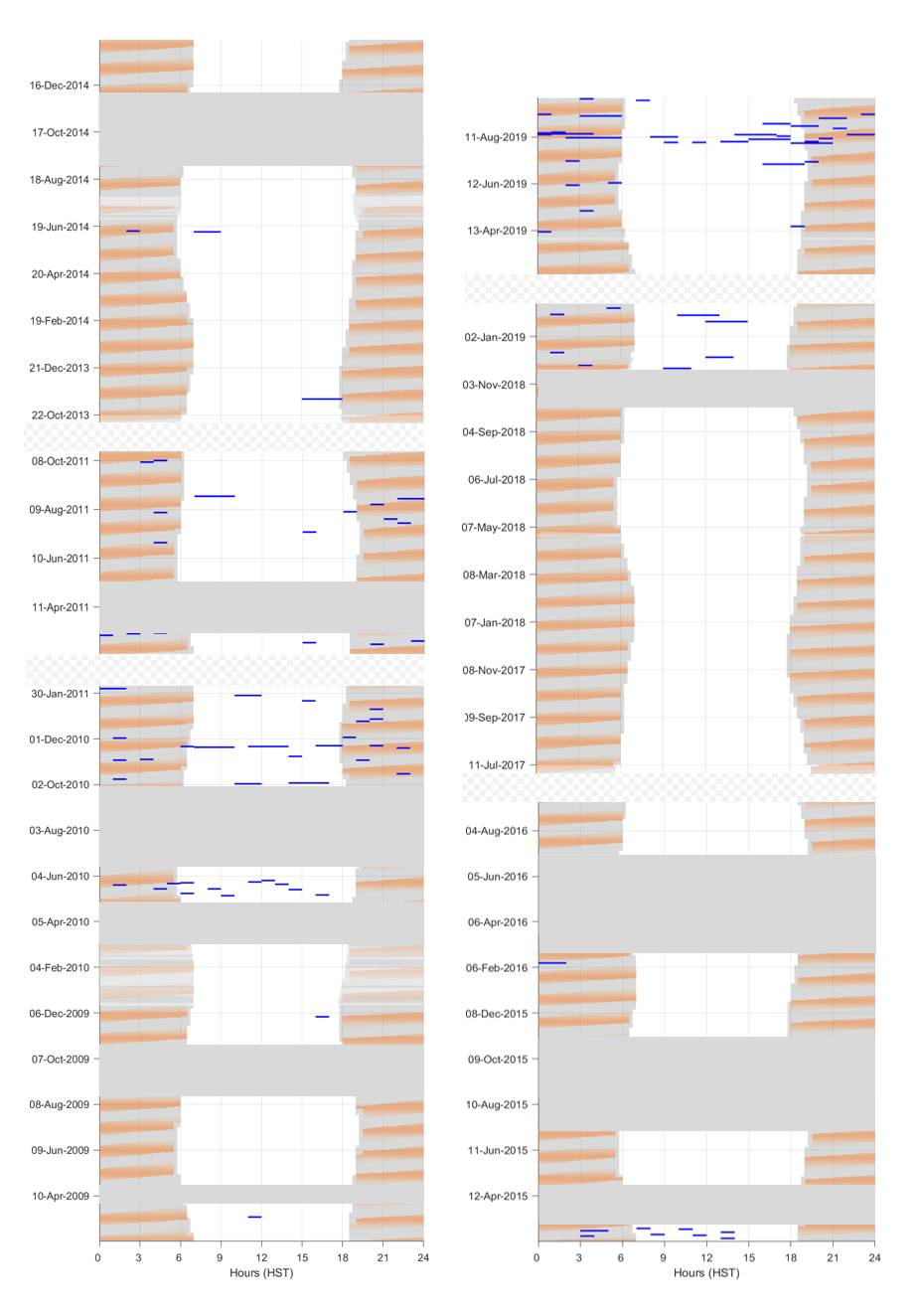
**

**Figure S3.24. Diel/lunar timeseries- False killer whale (alternate) at Hawaiʻi.** Hours of presence for false killer whales at Hawaiʻi (blue) for the precision-modified timeseries instead of the manually-edited timeseries. Date is shown on the y-axis, and hour of day on the x-axis. Lunar illumination (orange shading), nighttime hours (grey shading), times of no effort (dark gray boxes), and large time gaps (hashed gray boxes) are drawn.
